# Global, regional, and national burden of clavicle, scapula, or humerus fracture in 204 countries and territories, 1990 to 2021: A systematic analysis from the Global Burden of Disease Study 2021

**DOI:** 10.1097/MD.0000000000048862

**Published:** 2026-05-22

**Authors:** Xiaoyang Sun, Teng Yao, Fengzhen Wang

**Affiliations:** a Department of Orthopedics, Ningbo No. 6 Hospital, Ningbo, Zhejiang, China; b Ningbo Clinical Research Center for Orthopedics, Sports Medicine & Rehabilitation, Ningbo, Zhejiang, China; c Department of Orthopaedic Surgery, Sir Run Run Shaw Hospital, Zhejiang University School of Medicine, Hangzhou, Zhejiang, China; d Key Laboratory of Musculoskeletal System Degeneration and Regeneration Translational Research of Zhejiang Province, Hangzhou, Zhejiang, China; e Department of Radiology, Zhejiang Hospital, Hangzhou, Zhejiang, China.

**Keywords:** clavicle, Global Burden of Disease, humerus fracture, incidence, prevalence, scapula, YLDs

## Abstract

Clavicle, scapula, or humerus fracture represent a major public health challenge, yet their epidemiology and health burden remain poorly characterized. Using the Global Burden of Disease 2021 framework, we quantified incidence, prevalence, and years lived with disability (YLDs) of clavicle, scapula, or humerus fractures across 204 countries from 1990 to 2021. Estimates were stratified by age, sex, region, and socio-demographic index (SDI), and reported with 95% uncertainty intervals (UIs) from Bayesian models. Globally, the age-standardized incidence rate (ASIR) declined from 276.68 (95% UI: 219.06–346.71) per 100,000 person-years in 1990 to 220.94 (95% UI: 172.60–280.99) per 100,000 person-years in 2021, with an estimated annual percentage change of –0.78% (95% UI: –0.82% to –0.73%). High SDI regions showed the greatest ASIR (300.30 per 100,000 person-years), age-standardized prevalence rate (112.03 per 100,000 person-years), and age-standardized YLDs rate (3.72 per 100,000 person-years). In 2021, Eastern Europe had the highest ASIR (465.06 per 100,000 person-years), while Australasia led in age-standardized prevalence rate (151.42 per 100,000 person-years) and age-standardized YLDs rate (5.04 per 100,000 person-years). China ranked first in absolute prevalence (1.21 million, 95% UI: 1.04–1.39 million) and YLDs (40,235, 95% UI: 24,471–61,196). Despite declines in age-standardized rates, absolute cases increased substantially, highlighting demographic and regional disparities. Priority populations for targeted prevention include women aged ≥60 years and residents of high-SDI regions, as these strata accounted for the highest burden across incidence/prevalence/YLD rates.

## Graphical abstract

**Figure:**
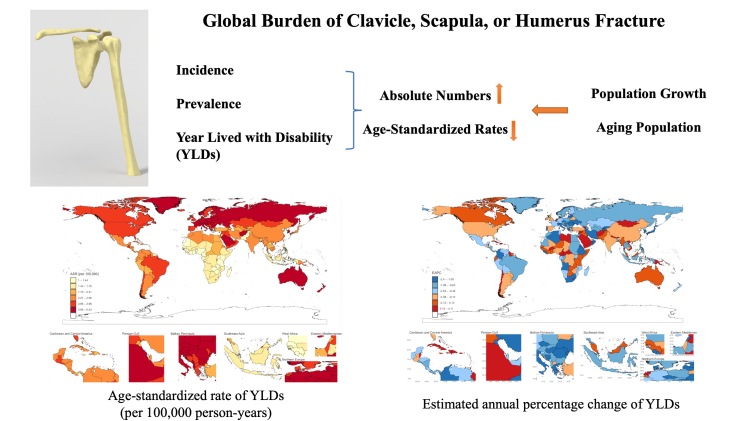

## 1. Introduction

Fractures represent a major global public health concern, contributing substantially to disability, healthcare utilization, and socioeconomic burden worldwide. In 2019 alone, an estimated 178 million new fractures occurred globally, resulting in approximately 25.8 million years lived with disability (YLDs) and reflecting a marked increase since 1990 driven largely by population growth and aging.^[1]^ Upper-extremity fractures constitute a large proportion of the overall fracture burden and include injuries involving the clavicle, scapula, and humerus. These fractures arise from diverse mechanisms such as falls, traffic accidents, and sports injuries and affect individuals across the lifespan.

Clavicle fractures are among the most common skeletal injuries, accounting for up to 10% of all fractures and occurring particularly frequently in children and young adults.^[2]^ Proximal humerus fractures are also common, representing approximately 4 to 10% of all fractures and occurring predominantly among older adults, often as fragility fractures related to osteoporosis.^[3]^ In contrast, scapular fractures are relatively uncommon but are frequently associated with high-energy trauma and complex injury patterns. Despite their clinical importance, the global epidemiological characteristics of fractures of the clavicle, scapula, and humerus remain incompletely characterized. Previous studies have primarily focused on specific fracture types or regional populations,^[4,5]^ while comprehensive analyses of their global burden, temporal trends, and demographic patterns remain limited. The Global Burden of Disease (GBD) study provides a unique framework for systematically quantifying the incidence, prevalence, and YLDs associated with these fractures across countries and over time.

Therefore, this study aimed to systematically evaluate the global burden of the clavicle, scapula, or humerus fracture (CSHF) using data from the GBD 2021 study. Specifically, we estimated incidence, prevalence, and YLDs across 204 countries and territories, examined temporal trends (1990–2021) to identify changing patterns over time, and characterized age- and sex-specific profiles to highlight priority populations for prevention and care. We also mapped geographic heterogeneity across GBD regions and evaluated socio-demographic gradients and inequality^[6]^ across the socio-demographic index (SDI) spectrum using complementary measures to inform equitable prevention strategies. Finally, we projected burden over the next 15 years to anticipate future healthcare and rehabilitation needs.

## 2. Materials and methods

### 2.1. Data source

The GBD Study 2021 (GBD 2021), led by the Institute for Health Metrics and Evaluation at the University of Washington, is a comprehensive global health initiative that systematically assessed the health burden of 371 diseases and injuries by integrating multi-source data from 204 countries and territories spanning 1990 to 2021.^[7]^ This data included census records, hospital registries, epidemiological surveys, and published literature. The study quantified core metrics such as incidence, prevalence, mortality, and disability-adjusted life-years, with stratified analyses by age, sex, geographic regions, and the SDI. SDI is a composite measure of development based on income per capita, educational attainment, and total fertility rate (0–1 scale) as defined in the GBD study. Data quality weighting and uncertainty intervals were incorporated to enhance the reliability of findings, providing high-resolution, evidence-based guidance for global public health decision-making.

### 2.2. Case definition

The GBD database systematically classifies injuries as health losses caused by external factors, including accidental injuries (traffic accidents, falls), intentional injuries (self-harm/interpersonal violence), and collective violence. Injury types and mechanisms are categorized based on International Classification of Diseases (ICD)-10 codes (V01–Y89). Fractures of the clavicle (ICD-10: S42.0), scapula (S42.1), and humerus (S42.2–S42.4) are defined as cases involving traumatic disruption of bone continuity, confirmed radiologically or clinically, and exclude pathological fractures (those directly caused by tumors or osteoporosis) and periprosthetic fractures. Disease classification adheres to the GBD etiology coding framework, ensuring cross-regional comparability through standardized mapping of clinical diagnostic data to the global disease classification system. In the GBD injury framework, when multiple fractures occur in a single injury event, only the most severe fracture site (based on disability weights) is assigned to that event. Thus, concomitant less severe fractures may not be counted in site-specificincidence estimates, potentially leading to underestimation.

### 2.3. Core metrics

Incidence rate refers to the proportion of new disease cases within a specific population during a defined period (typically 1 year), reflecting the risk of disease occurrence. Prevalence rate represents the total number of existing cases (including both new and unresolved cases) in a population at a specific time point or period, indicating the cumulative disease burden influenced by both incidence and disease duration. Years lived with disability (YLDs) quantifies the loss of healthy life years due to nonfatal health impairments. Calculated by combining disability weights (ranging from 0, indicating full health, to 1, equivalent to death) with the duration of disability, YLDs in the GBD framework integrate globally standardized disability weight tables and corrections using the DisMod-MR model to ensure precise assessment of long-term impacts on quality of life.

### 2.4. Model framework

The GBD study employs a hierarchical Bayesian modeling framework that integrates multi-source heterogeneous global data. It addresses geographic and temporal data gaps using spatiotemporal Gaussian process regression and unifies epidemiological parameter estimation through DisMod-MR 2.1. Covariate adjustments incorporate the SDI and risk factor stratification. The framework generates 95% uncertainty intervals (UIs) via iterative sampling to quantify potential biases arising from data quality, diagnostic heterogeneity, and model assumptions. Final outputs include age–sex–region standardized disease burden metrics. The Bayesian Age–Period-Cohort (BAPC) model was prioritized over simpler time-series approaches due to its capacity to simultaneously disentangle age, period, and cohort effects – critical for projecting disease burden in evolving populations with demographic transitions.

### 2.5. Statistical analysis

All metrics (incidence, prevalence, and YLDs rates) were directly age-standardized using the GBD 2021 global standard population structure, with results expressed as per 100,000 person–years to eliminate confounding effects of population age composition on cross-regional and temporal comparisons. The formula is as follows:


$$\frac{\overset{A}{\underset{i=1}{\sum }}a_{i}w_{i}}{\overset{A}{\underset{i=1}{\sum }}w_{i}}$$

Based on the GBD hierarchical Bayesian model, the Markov Chain Monte Carlo method was utilized to perform 1000 iterative samplings of model parameters, generating 95% uncertainty intervals (95% UIs) for all metrics. These intervals comprehensively account for the impacts of data source heterogeneity, model assumption biases, and sampling errors on estimates, with interval ranges defined by the 2.5th to 97.5th percentiles.

Estimated annual percentage change (EAPC) was used to quantify temporal trends in disease burden metrics from 1990 to 2021, calculated by fitting a log-linear regression model. The formula is as follows:


y=α+βx+∈

$$EAPC=100\;\%\;\times \left(e^{\beta }-1\right)$$

Pearson correlation analyses were conducted to assess linear associations between age-standardized incidence rates/prevalence rates/YLDs rates and SDI across GBD 21 regions/204 countries. Variables were inspected for approximate normality; when skewed, log-transformation was applied prior to analysis. We report correlation coefficients (*r*) and 2-sided *P*-values. As a robustness check, Spearman rank correlations were additionally performed to evaluate monotonic associations.

### 2.6. Health inequality analysis

Countries were ranked by their SDI from lowest to highest and assigned normalized ranks (0 for the most disadvantaged, 1 for the most advantaged). The Slope Index of Inequality (SII) was calculated using a weighted least squares linear regression model to measure the absolute gradient association between health metrics and standardized ranks, reflecting the disparity in disease burden across socioeconomic development levels. For the concentration index (ConI), countries were similarly ranked by SDI, and cumulative percentages of health burden and population were computed. A health concentration curve (analogous to a Lorenz curve) was plotted, with the confidence interval (CI) derived from the area between the curve and the line of equality. The CI ranges from −1 to 1, where a negative value indicates that the health burden is concentrated among populations with lower SDI.

### 2.7. Projection of epidemiologic trends

Projections were generated using the BAPC model under the core assumption that current epidemiological trends will persist. The model incorporates age effects (biological risk), period effects (environmental/policy changes), and cohort effects (birth generation influences). Posterior distributions of parameters were generated via Markov Chain Monte Carlo simulation to derive projected values and their 95% UI.

### 2.8. Ethics approval and consent to participate

Ethics approval was not required for this study due to the analysis of anonymized and publicly available data.

## 3. Results

### 3.1. Global burden of CSHF

In 2021, CSHF globally resulted in 17.59 million new cases (95% UI: 13.73–22.32 million), 6.63 million prevalent cases (95% UI: 5.72–7.67 million), and 219,536 YLDs (95% UI: 136,009–334,653). The global age-standardized incidence rate (ASIR) was 220.94 cases per 100,000 person-years (95% UI: 172.60–280.99 per 100,000 person-years), with an age-standardized prevalence rate (ASPR) of 80.74 per 100,000 person-years (95% UI: 69.55–93.69 per 100,000 person-years) and age-standardized YLDs rate (ASDR) of 2.67 per 100,000 person-years (95% UI: 1.65–4.08 per 100,000 person-years). Despite rising absolute numbers of incidence cases, prevalent cases, and YLDs, all age-standardized global metrics exhibited significant declines (EAPC of ASIR = −0.78%, 95% CI: −0.82 to −0.73%; EAPC of ASPR = −0.71%, 95% CI: −0.75 to −0.67%; EAPC of ASDR = −0.73%, 95% CI: −0.77 to −0.68%). Males showed higher ASIR, while ASPR and ASDR were similar between sexes. However, females surpassed males in prevalent cases and YLDs counts globally over the past decade (Table 1 and see Figure S1).

**Table 1:** Incidence, prevalence and YLDs of fracture of clavicle, scapula, or humerus and their estimated annual percentage change (EAPC) from 1990 to 2021 globally, across different SDI regions, and in 21 regions.

Location	Incidence					Prevalence					YLDs				
	Number (95% UI)		ASR per 100,000 (95% UI)		EAPC (95% CI)	Number (95% UI)		ASR per 100,000 (95% UI)		EAPC (95% CI)	Number (95% UI)		ASR per 100,000 (95% UI)		EAPC (95% CI)
	1990	2021	1990	2021		1990	2021	1990	2021		1990	2021	1990	2021	
Global	14,759,852 (11,759,957–18,546,936)	17,588,524 (13,726,696–22,315,371)	276.68 (219.06–346.71)	220.94 (172.6–280.99)	−0.78 (−0.82 to −0.73)	4,481,697 (3,835,271–5,223,650)	6,629,904 (5,723,414–7,672,986)	97.88 (84.81–112.4)	80.74 (69.55–93.69)	−0.71 (−0.75 to −0.67)	150,879 (91,948–229,738)	219,536 (136,009–334,653)	3.26 (2.01–4.97)	2.67 (1.65–4.08)	−0.73 (−0.77 to −0.68)
High SDI	3,273,472 (2,522,640–4,218,347)	3,585,847 (2,661,718–4,767,154)	367.97 (283.21–479.94)	300.3 (224.99–397.11)	−0.72 (−0.75 to −0.68)	1,302,095 (1,132,856–1,498,592)	1,852,741 (1,605,951–2,123,090)	132.08 (113.44–153.08)	112.03 (96.21–131.38)	−0.58 (−0.6 to −0.55)	43,268 (25,891–65,219)	60,285 (36,767–90,158)	4.41 (2.65–6.66)	3.72 (2.23–5.68)	−0.6 (−0.62 to −0.57)
High-middle SDI	3,647,766 (2,900,467–4,635,278)	3,566,452 (2,755,861–4,629,127)	338.86 (268.6–431.68)	272.65 (211.8–357.85)	−0.91 (−0.99 to −0.83)	1,175,990 (1,005,612–1,363,370)	1,443,926 (1,258,676–1,656,630)	114.63 (98.55–132.34)	90.36 (76.97–105.44)	−0.99 (−1.08 to −0.9)	39,577 (24,142–60,808)	47,886 (29,695–73,225)	3.84 (2.35–5.88)	3.02 (1.84–4.68)	−0.99 (−1.08 to −0.9)
Middle SDI	4,126,076 (3,315,339–5,170,284)	5,017,542 (3,932,299–6,440,364)	233.12 (186.74–291.3)	204.04 (159.6–264.58)	−0.34 (−0.4 to −0.28)	1,091,565 (910,268–1,281,644)	1,770,730 (1,520,187–2,042,053)	76.09 (65.22–87.66)	70.2 (60.03–81.31)	−0.31 (−0.39 to −0.22)	37,108 (22,083–56,946)	59,001 (36,018–89,437)	2.55 (1.55–3.86)	2.33 (1.43–3.55)	−0.32 (−0.4 to −0.24)
Low-middle SDI	2,601,331 (2,068,649–3,265,860)	3,513,418 (2,779,420–4,485,177)	224.9 (179.35–282.98)	186.73 (146.62–237.42)	−0.71 (−0.81 to −0.62)	659,001 (547,680–784,188)	1,080,511 (914,318–1,266,249)	74.57 (63.77–86.35)	66.98 (57.12–77.42)	−0.43 (−0.5 to −0.37)	22,315 (13,359–34,350)	36,072 (21,761–55,269)	2.47 (1.51–3.71)	2.2 (1.35–3.37)	−0.45 (−0.51 to −0.39)
Low SDI	1,092,420 (834,563–1,432,298)	1,888,086 (1,467,984–2,418,326)	207.82 (161.84–268.69)	170.7 (134.3–212.36)	−0.48 (−0.71 to −0.25)	247,285 (193,688–316,155)	475,675 (383,322–578,098)	63.62 (53.32–75.75)	59.39 (50.42–69.62)	−0.21 (−0.32 to −0.1)	8418 (5126–13,441)	16,082 (9937–25,078)	2.11 (1.29–3.29)	1.96 (1.23–2.96)	−0.22 (−0.33 to −0.11)
Region															
Andean Latin America	101,890 (80,734–129,067)	135,650 (109,047–171,722)	249.29 (200.64–310.51)	201.66 (162.32–255.78)	−0.48 (−0.6 to −0.37)	23,013 (18,473–28,052)	38,697 (32,801–45,245)	68.34 (57.38–81.46)	59.89 (51.02–69.65)	−0.34 (−0.4 to −0.27)	789 (477–1251)	1308 (771–2041)	2.32 (1.4–3.61)	2.02 (1.19–3.13)	−0.34 (−0.41 to −0.28)
Australasia	97,247 (74,276–128,795)	135,434 (100,124–183,021)	489.71 (370.32–655.19)	438.22 (323.61–609.1)	−0.3 (−0.41 to −0.19)	34,493 (29,397–40,315)	62,610 (53,592–72,222)	162.46 (137–192.21)	151.42 (126.48–178.78)	−0.12 (−0.22 to −0.02)	1150 (690–1750)	2047 (1231–3105)	5.43 (3.23–8.33)	5.04 (2.98–7.73)	−0.14 (−0.24 to −0.04)
Caribbean	74,540 (61,181–92,648)	109,519 (88,709–136,185)	206.44 (169.13–256.85)	231.42 (187.61–287.29)	0.3 (−0.32 to 0.93)	20,279 (17,140–24,217)	37,443 (32,423–43,253)	64.56 (55.64–75.17)	73.97 (63.41–85.97)	0.45 (0.1 to 0.81)	688 (410–1070)	1242 (781–1914)	2.17 (1.31–3.33)	2.46 (1.54–3.79)	0.43 (0.06 to 0.79)
Central Asia	241,750 (194,084–306,394)	250,673 (202,095–317,114)	329.11 (266.06–416.45)	259.73 (209.03–328.36)	−0.96 (−1.16 to −0.77)	58,631 (48,590–70,901)	68,299 (57,459–80,966)	91.89 (77.9–108.73)	73.62 (62.36–86.91)	−0.83 (−0.94 to −0.72)	2006 (1178–3149)	2320 (1392–3619)	3.12 (1.87–4.83)	2.49 (1.5–3.87)	−0.84 (−0.95 to −0.73)
Central Europe	747,164 (585,208–957,245)	504,032 (380,758–647,523)	603.98 (473.8–772.68)	452.67 (347.54–594.15)	−1.11 (−1.18 to −1.04)	234,491 (199,746–274,553)	199,495 (173,682–230,073)	179.19 (151.66–212.39)	131.16 (109.52–156.69)	−1.17 (−1.23 to −1.11)	7853 (4705–12,158)	6589 (3981–10,172)	6 (3.59–9.34)	4.41 (2.62–6.93)	−1.16 (−1.22 to −1.09)
Central Latin America	641,343 (509,004–820,692)	622,268 (494,265–808,456)	364.85 (290.73–471.25)	247.21 (195.75–321.83)	−0.69 (−0.93 to −0.44)	155,036 (125,750–189,884)	193,093 (164,485–225,028)	111.94 (94.68–131.14)	76.21 (64.85–89.22)	−0.78 (−0.98 to −0.58)	5280 (3149–8305)	6484 (3905–9867)	3.76 (2.28–5.77)	2.56 (1.54–3.9)	−0.78 (−0.98 to −0.58)
Central Sub-Saharan Africa	87,153 (71,211–108,870)	177,374 (144,647–216,305)	147.5 (121.02–181.25)	128.79 (106.17–156.16)	−1.33 (−2.09 to −0.56)	20,499 (16,656–25,101)	45,593 (37,215–55,953)	48.15 (41.3–56.07)	46.67 (39.52–55.22)	−0.56 (−0.95 to −0.18)	699 (421–1099)	1545 (963–2379)	1.61 (0.98–2.47)	1.54 (0.98–2.33)	−0.59 (−0.99 to −0.19)
East Asia	2,277,550 (1,786,456–2,861,674)	3,000,506 (2,301,322–3,942,348)	186.28 (146.3–234.03)	196.75 (152.43–258.02)	−0.23 (−0.56 to 0.11)	691,379 (584,290–813,178)	1,237,024 (1,065,871–1,418,407)	66.29 (56.89–76.57)	69.04 (58.65–80.43)	−0.21 (−0.52 to 0.1)	23,475 (13,905–35,644)	41,095 (25,011–62,445)	2.22 (1.33–3.37)	2.3 (1.38–3.54)	−0.23 (−0.54 to 0.07)
Eastern Europe	1,323,032 (1,059,909–1,687,610)	919,042 (725,507–1,192,239)	595.99 (477.16–756.08)	465.06 (370.02–602.03)	−1.12 (−1.39 to −0.85)	417,673 (358,032–486,029)	342,239 (297,897–391,167)	172.11 (146.05–203.34)	135.51 (115.04–159.37)	−1.1 (-1.4 to −0.81)	14,052 (8363–21,581)	11,399 (6943–17,475)	5.81 (3.44–9)	4.57 (2.72–7.07)	−1.1 (−1.4 to −0.81)
Eastern Sub-Saharan Africa	510,741 (338,643–786,485)	524,640 (411,751–655,300)	237.17 (166.13–350.14)	122.77 (99.7–150.24)	−1.62 (−2.11 to −1.12)	100,792 (70,846–147,649)	131,220 (103,328–162,690)	60.83 (47.26–81.35)	43.64 (36.04–53.41)	−0.87 (−1.11 to −0.63)	3463 (1922–6011)	4455 (2695–7101)	2.05 (1.2–3.37)	1.45 (0.91–2.19)	−0.9 (−1.15 to −0.65)
High-income Asia Pacific	605,299 (465,927–791,829)	458,934 (338,799–610,025)	349.83 (268.61–458.12)	238.68 (177.4–320.84)	−1.42 (−1.53 to −1.31)	222,869 (193,468–258,234)	264,749 (231,189–301,810)	119.77 (102.56–140.17)	85.48 (73.09–100.77)	−1.27 (−1.38 to −1.15)	7500 (4501–11,478)	8667 (5246–13,108)	4.03 (2.42–6.14)	2.87 (1.7–4.44)	−1.28 (−1.39 to −1.16)
High-income North America	912,468 (702,512–1,176,089)	1,148,972 (851,253–1,514,209)	317.79 (244.44–409.98)	262.46 (198.85–339.72)	−0.73 (−0.9 to −0.56)	374,898 (325,213–430,516)	636,071 (544,810–737,513)	118.05 (101.4–136.25)	113.05 (97.3–131.09)	−0.18 (−0.31 to −0.06)	12,431 (7529–18,752)	20,509 (12,645–30,545)	3.94 (2.37–5.92)	3.7 (2.27–5.56)	−0.25 (−0.37 to −0.12)
North Africa and Middle East	1,111,637 (905,649–1,347,726)	1,784,657 (1,432,284–2,228,103)	305.16 (249.74–370.56)	281.11 (225.29–350.68)	0.18 (−0.01 to 0.36)	266,560 (217,663–325,565)	484,094 (400,018–583,673)	91.07 (76.87–108.3)	83.33 (69.66–99.19)	−0.08 (−0.16 to 0)	9099 (5596–14,055)	16,368 (10,246–24,786)	3.07 (1.91–4.67)	2.79 (1.76–4.23)	−0.09 (−0.17 to 0)
Oceania	9396 (7532–11,730)	23,065 (18,179–28,806)	146.36 (116.72–181.57)	171.49 (134.03–214.05)	0.13 (−0.25 to 0.52)	2395 (1999–2845)	6363 (5374–7529)	51.66 (45.11–59.49)	61.65 (53.52–71.19)	0.36 (0.17 to 0.55)	82 (48–127)	215 (125–333)	1.72 (1.05–2.64)	2.04 (1.22–3.11)	0.35 (0.15 to 0.54)
South Asia	2,342,621 (1,804,835–3,007,247)	3,556,466 (2,722,017–4,661,991)	225.16 (173.86–290.94)	198.19 (150.91–262.23)	−0.53 (−0.62 to −0.43)	611,681 (508,185–732,909)	1,172,587 (982,811–1,385,718)	79.09 (66.56–92.21)	75.82 (64.31–88.27)	−0.24 (−0.31 to −0.18)	20,606 (12,113–31,928)	38,818 (23,479–59,820)	2.59 (1.58–3.93)	2.47 (1.5–3.77)	−0.26 (−0.32 to −0.19)
Southeast Asia	963,227 (789,306–1,173,201)	1,131,485 (916,362–1,416,361)	202.14 (165.98–247.64)	162.9 (131.33–203.89)	−0.69 (−0.9 to −0.48)	245,495 (204,041–294,303)	365,586 (314,278–423,980)	65.1 (56.03–75.43)	55.18 (47.46–63.77)	−0.58 (−0.68 to −0.49)	8364 (5063–12,925)	12,275 (7591–18,754)	2.18 (1.34–3.29)	1.84 (1.14–2.8)	−0.6 (−0.7 to −0.5)
Southern Latin America	146,082 (110,317–191,415)	193,767 (147,748–255,338)	292.94 (221.23–382.81)	290.29 (220.59–383.56)	0.01 (−0.15 to 0.16)	46,793 (39,497–55,630)	72,930 (62,808–85,181)	98.09 (83.25–115.65)	96.59 (81.54–114.4)	−0.02 (−0.14 to 0.1)	1580 (929–2459)	2433 (1463–3780)	3.3 (1.95–5.13)	3.24 (1.94–5.09)	−0.02 (−0.14 to 0.1)
Southern Sub-Saharan Africa	101,830 (82,560–125,454)	113,616 (92,351–139,585)	191.64 (155.09–236.46)	137.53 (111.8–169.02)	−1.24 (−1.36 to −1.12)	27,867 (23,627–32,988)	34,108 (29,356–39,966)	66.76 (58.54–76.52)	46.46 (40.65–53.6)	−1.35 (−1.5 to −1.21)	947 (564–1446)	1150 (702–1740)	2.24 (1.36–3.38)	1.55 (0.96–2.34)	−1.36 (−1.51 to −1.22)
Tropical Latin America	612,145 (474,277–791,400)	670,036 (529,211–862,647)	383.56 (300.47–500.67)	293 (230.75–379.54)	−0.79 (−0.94 to −0.63)	153,998 (125,553–186,010)	221,499 (188,875–258,484)	116.61 (98.69–137.63)	91.45 (77.4–107.33)	−0.77 (−0.89 to −0.65)	5235 (3049–8209)	7389 (4435–11,359)	3.91 (2.35–6.02)	3.06 (1.82–4.7)	−0.78 (−0.89 to −0.66)
Western Europe	1,595,411 (1,183,846–2,100,219)	1,551,777 (1,083,378–2,116,632)	407.39 (301.06–540.06)	333.35 (234.52–457.12)	−0.68 (−0.76 to −0.6)	712,962 (621,689–822,024)	878,422 (759,566–1,016,419)	150.74 (129.56–175.62)	125.76 (106.73–149.54)	−0.6 (−0.67 to −0.53)	23,536 (14,099–35,294)	28,527 (17,511–42,789)	5.02 (2.98–7.59)	4.18 (2.48–6.4)	−0.6 (−0.67 to −0.54)
Western Sub-Saharan Africa	257,326 (210,829–312,899)	576,613 (471,824–713,232)	127.43 (105.22–154.71)	118.6 (97.1–146.74)	−0.31 (−0.43 to −0.18)	59,893 (49,295–73,001)	137,784 (113,978–165,293)	40.98 (35.51–47.32)	40.21 (34.79–46.17)	−0.12 (−0.19 to −0.05)	2045 (1203–3182)	4700 (2815–7349)	1.37 (0.84–2.09)	1.34 (0.83–2.02)	−0.13 (−0.19 to −0.06)

ASR = age-standardized rate, CI = confidence interval, EAPC = estimated annual percentage change, SDI = socio-demographic index, UI = uncertainty interval, YLDs = years lived with disability.

### 3.2. Burden of CSHF in 5 SDI regions

The burden of CSHF exhibited a pronounced gradient aligned with socioeconomic development, showing a strong positive correlation with SDI. High-SDI regions had the highest ASIR (300.30 per 100,000 person-years, 95% UI: 224.99–397.11), ASPR (112.03 per 100,000 person-years, 95% UI: 96.21–131.38), and ASDR (3.72 per 100,000 person-years, 95% UI: 2.23–5.68), which were 75.9%, 88.6%, and 89.8% higher than those in low-SDI regions (incidence: 170.70; prevalence: 59.39; YLDs: 1.96 per 100,000 person-years). All SDI quintile regions demonstrated significant declines in age-standardized rates of fracture burden during 1990 to 2021. The largest reductions occurred in the high-middle SDI region, with EAPCs of −0.91% (ASIR), −0.99% (ASPR), and −0.99% (ASDR) (Table 1 and see Figure S2).

### 3.3. Regional burden of CSHF

**Figure 1.**
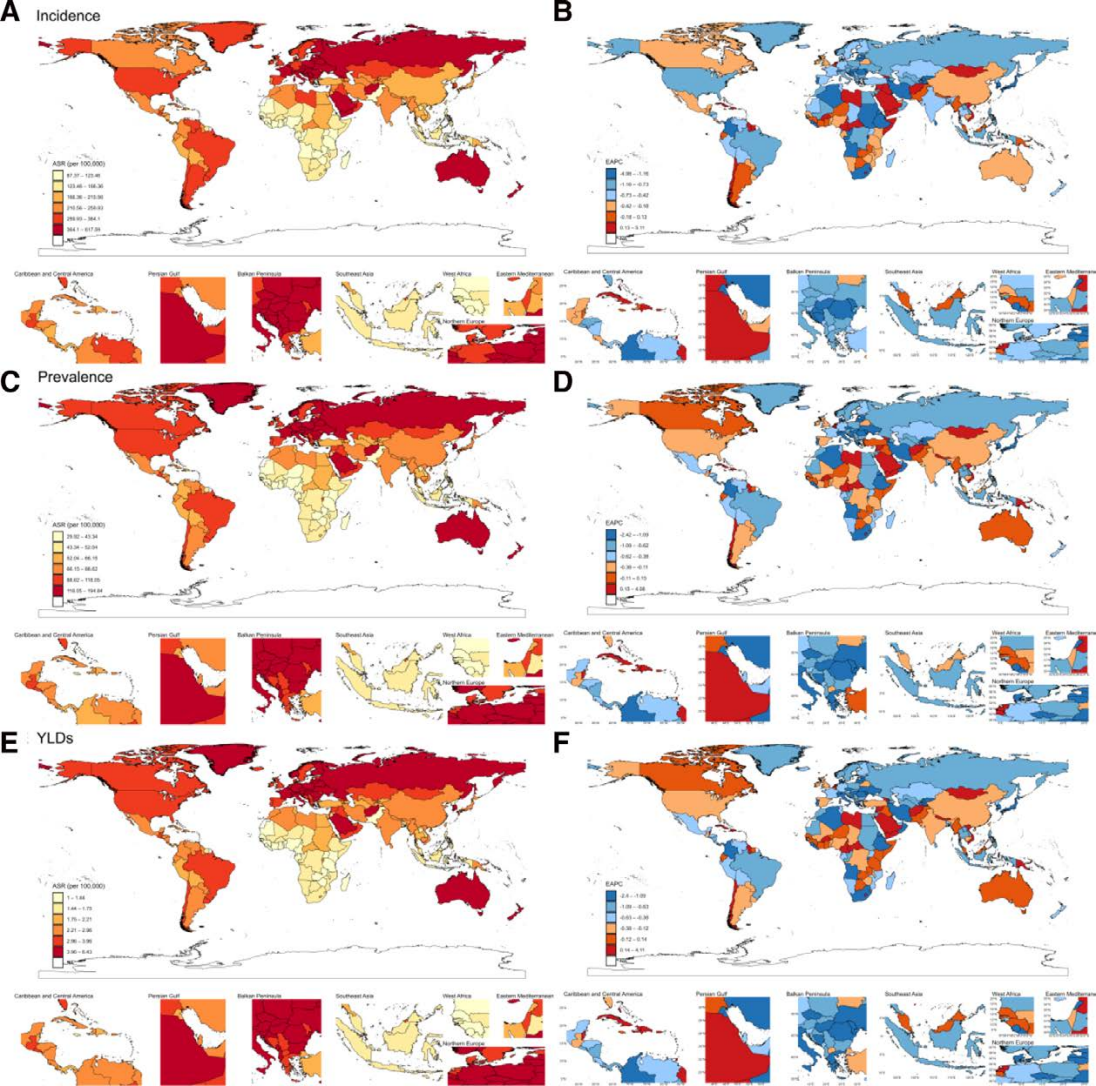
Age-standardized rates of incidence, prevalence, and YLDs, along with their estimated annual percentage change (EAPC) from 1990 to 2021, in fracture of clavicle, scapula, or humerus across countries. (A) and (B) Incidence. (C) and (D) Prevalence. (E) and (F) YLDs. YLDs = years lived with disability.

**Figure 2.**
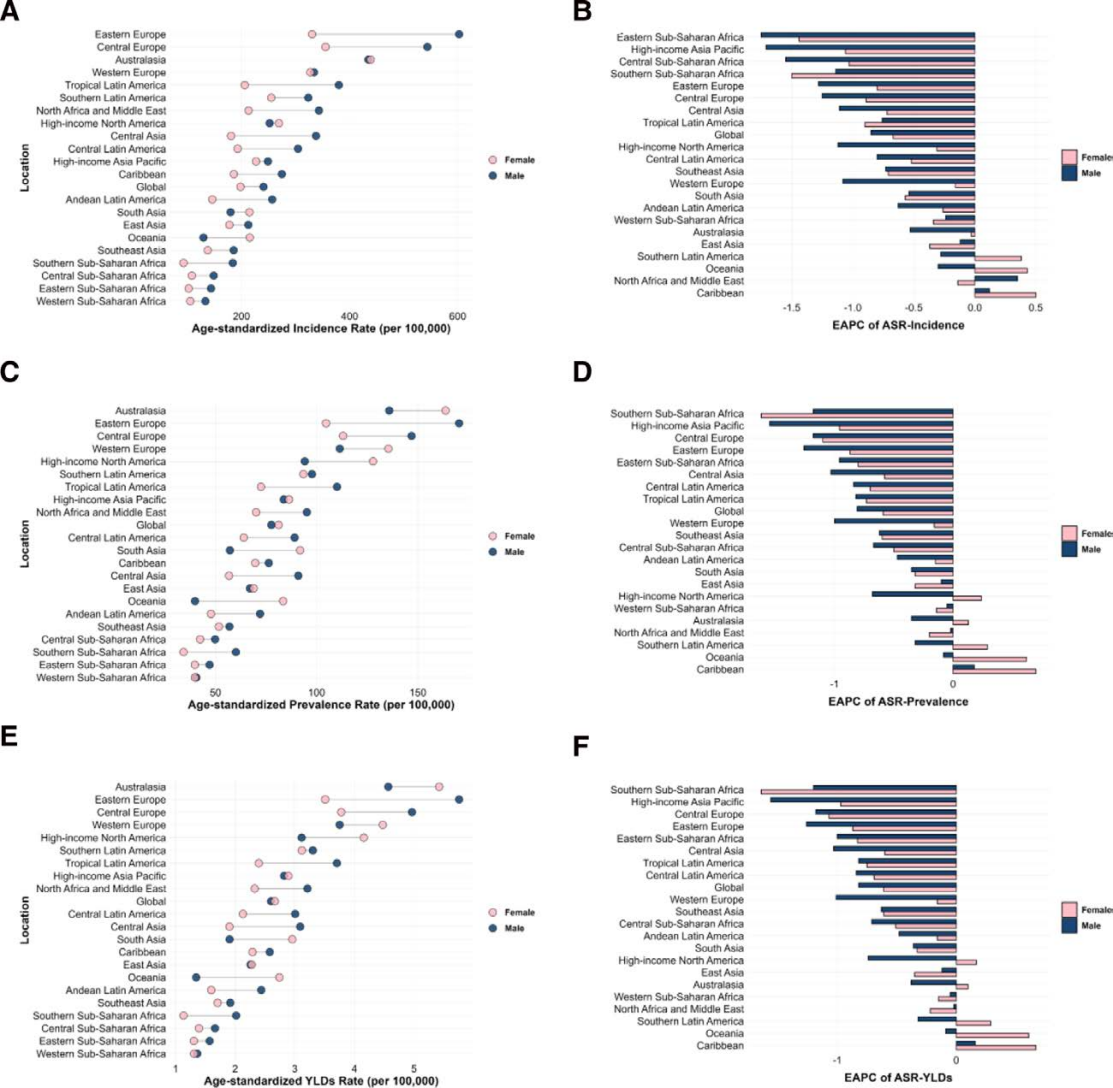
Age-standardized rates of incidence, prevalence, and YLDs, along with their estimated annual percentage change (EAPC) from 1990 to 2021, in fracture of clavicle, scapula, or humerus across 21 regions. (A) and (B) Incidence; (C) and (D) Prevalence; (E) and (F) YLDs. YLDs = years lived with disability.

In 2021, the burden of CSHF exhibited marked regional disparities: Eastern Europe reported the highest ASIR (465.06 per 100,000 person-years, 95% UI: 370.02–602.03), while Australasia had the highest ASPR (151.42 per 100,000 person-years, 95% UI: 126.48–178.78) and ASDR (5.04 per 100,000 person-years, 95% UI: 2.98–7.73). Notably, Eastern Europe exhibited the second-highest global ASPR (135.51 per 100,000 person-years) and ASDR (4.57 per 100,000 person-years), closely following Australasia, confirming its substantial overall disease burden. From 1990 to 2021, Eastern subSaharan Africa achieved the largest decline in ASIR (EAPC: −1.62%, 95% CI: −2.11 to −1.12%), whereas Southern Sub-Saharan Africa showed the most significant reductions in ASPR (EAPC: −1.35%, 95% CI: −1.50 to −1.21%) and ASDR (EAPC: −1.36%, 95% CI: −1.51 to −1.22%). Conversely, Caribbean (EAPC of ASPR: 0.45%, EAPC of ASDR: 0.43%) and Oceania (EAPC of ASPR: +0.36%, EAPC of ASDR: 0.35%) displayed significant upward trends (*P* < .05) (Table 1 and Fig. 1). The sex-stratified age-standardized metrics and EAPC for 21 GBD regions were presented in Figure 2.

### 3.4. National heterogeneity in the burden of CSHF

In 2021, the burden of CSHF showed substantial country-level disparities. India reported the highest number of incidence cases (3.03 millions, 95% UI: 2.32–4.01 millions), while Afghanistan had the highest ASIR (368.72 per 100,000 person-years, 95% UI: 287.45–452.19). China ranked first globally in both prevalence cases (1.21 millions, 95% UI: 1.04–1.39 millions) and YLDs (40,235, 95% UI: 24,471–61,196). In contrast, Andorra exhibited the highest ASPR (194.84 per 100,000 person-years, 95% UI: 165.19–231.16) and ASDR (6.43 per 100,000 person-years, 95% UI: 3.80–9.66). The Syrian Arab Republic experienced the most significant increases in age-standardized metrics: ASIR (EAPC: 5.11%, 95% CI: 3.27–6.98%), ASPR (EAPC: 4.08%, 95% CI: 2.89–5.29%), and ASDR (EAPC: 4.11%, 95% CI: 2.89–5.34%) (see Table S1, and Fig. 1).

### 3.5. Age–sex distribution of global CSHF burden

The global burden of CSHF in 2021 exhibited a distinct age–sex distribution. Incidence cases peaked at 20 to 24 years (1.60 millions, 95% UI: 1.21–2.08 millions), while prevalence cases (503,427, 95% UI: 434,533–578,425) and YLDs (16,439, 95% UI: 9956–24,716) reached their highest levels at 65 to 69 years, following the peak of incidence cases. Incidence rates followed a bimodal pattern: an initial peak at 20 to 24 years (268.51 cases per 100,000 person-years), followed by a temporary decline, then a second peak at ≥95 years (694.69 cases per 100,000 person-years). Prevalence and YLDs rates increased monotonically with age. Males had higher rates and absolute numbers than females in populations <60 years, but this trend reversed in those ≥60 years. From 1990 to 2021, declines in age-standardized rates diminished with advancing age, and even shifted to rising trends in older populations (see Table S2 and Figure S3).

### 3.6. Relationships of the age-standardized incidence, prevalence, and YLDs rates of CSHF and their trends with SDI

**Figure 3.**
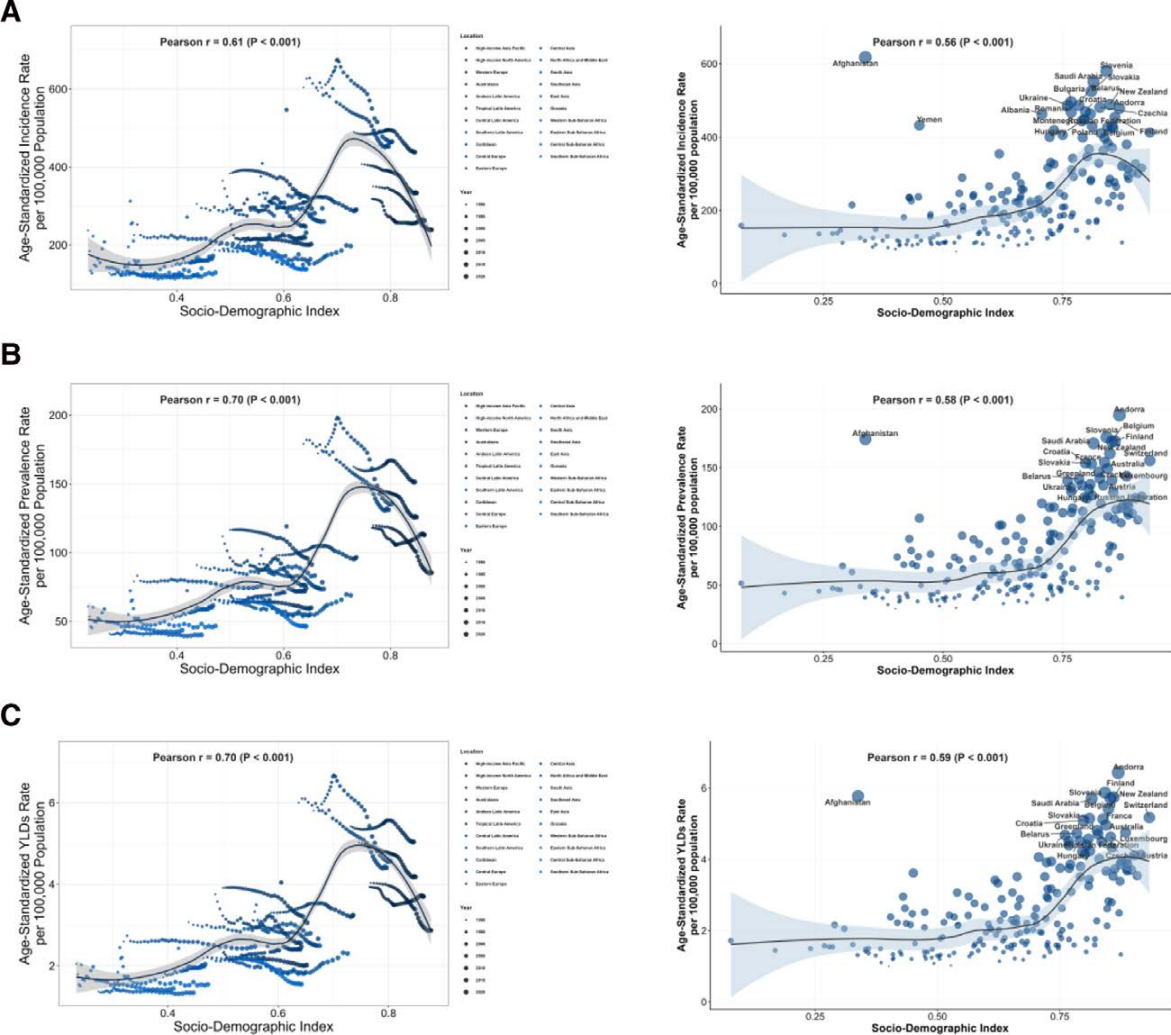
Burden of fracture of clavicle, scapula, or humerus across 21 regions and 204 countries by SDI. (A) Age-standardized incidence rate for 21 regions and 204 countries by SDI from 1990 to 2021. (B) Age-standardized prevalence rate for 21 regions and 204 countries by SDI from 1990 to 2021. (C) Age-standardized YLDs rate for 21 regions and 204 countries by SDI from 1990 to 2021. SDI = socio-demographic index; YLDs = years lived with disability.

At the 21 GBD regional level, ASIR (Pearson *R* = 0.61), ASPR (Pearson *R* = 0.70), and ASDR (Pearson *R* = 0.70) exhibited significantly positive associations with SDI (*P* < .001). This pattern was attenuated at the country level, though ASIR (Pearson *R* = 0.56), ASPR (0.58), and ASDR (0.59) remained positively correlated with national SDI (Fig. 3).

### 3.7. Evolution of health inequalities in CSHF burden

**Figure 4.**
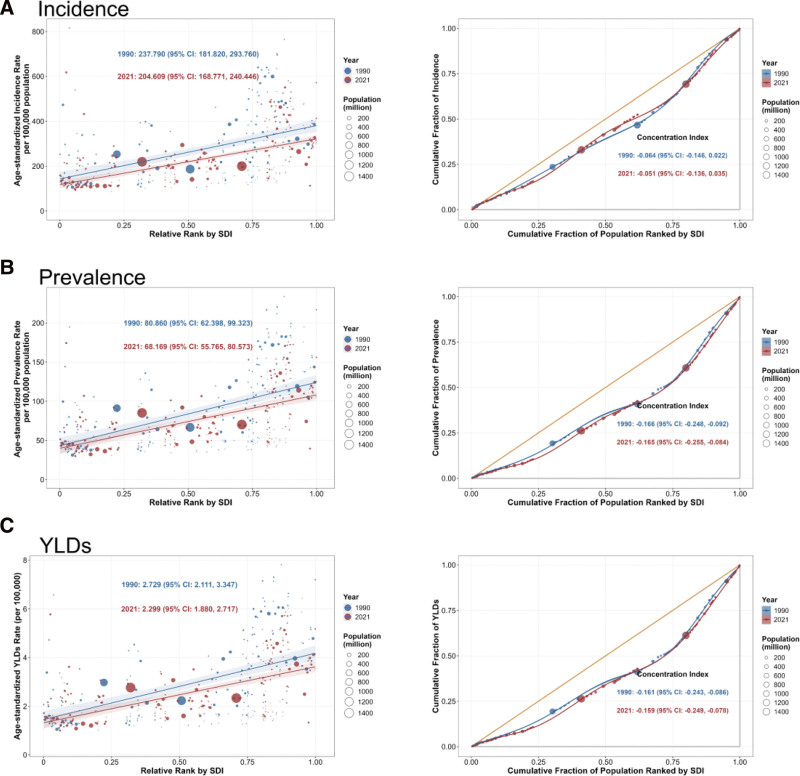
Slope index of inequality (left) and concentration index (right) curves for fracture of clavicle, scapula, or humerus in 1990 and 2021. (A) Incidence. (B) Prevalence. (C) YLDs. YLDs = years lived with disability.

Health inequality analysis reveals significant shifts in the association between fracture burden and socioeconomic development from 1990 to 2021. Incidence SII decreased from 237.79 per 100,000 person-years (95% CI: 181.82–293.76) to 204.61 per 100,000 person-years (95% CI: 168.77–240.45). Prevalence SII declined from 80.86 per 100,000 person-years to 68.17 per 100,000 person-years, and YLDs SII decreased from 2.73 per 100,000 person-years to 2.30 per 100,000 person-years (*P* < .01), indicating a weakening absolute impact of the SDI gradient on fracture risk. Incidence ConI increased from −0.064 (95% CI: −0.146 to 0.022) to −0.051 (95% CI: −0.136 to 0.035) but not statistically difference. Prevalence and YLDs ConIs were significantly negative, indicating a partial alleviation of the burden concentrated in low-SDI populations (see Table S3 and Fig. 4).

### 3.8. Projections of global burden of CSHF

**Figure 5.**
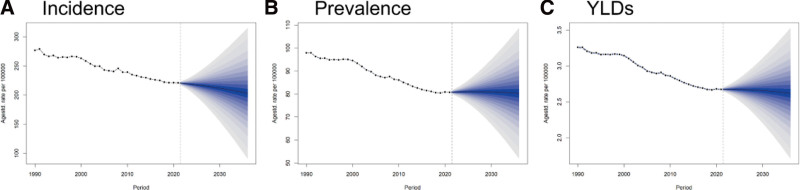
Trends and forecasts of the burden of fracture of clavicle, scapula, or humerus globally until 2036. (A) Incidence. (B) Prevalence. (C) YLDs. YLDs = years lived with disability.

Forecasts based on the BAPC model indicated complex dynamic trends in the global burden of CSHF from 2022 to 2036. Incidence rates is projected to decline, reaching 202.94 per 100,000 person-years (95% UI: 90.77–315.10) by 2036. Prevalence rates is projected to temporarily rise, peaking in 2027 at 81.01 per 100,000 person-years (95% UI: 72.82–89.20), followed by a downward trend to 80.29 per 100,000 person-years (95% UI: 51.85–108.72) by 2036. YLDs rates is projected to exhibit a gradual decline over the same period (see Table S4 and Fig. 5).

## 4. Discussion

Global epidemiological data from 2021 revealed contrasting trends in CSHF burden: while the absolute number of incident cases, prevalent cases, and YLDs showed consistent annual increases, the age-standardized rates for incidence, prevalence, and YLDs demonstrated progressive declines. This paradoxical pattern reflects the combined effects of global population growth and demographic aging.^[8–12]^ Notably, women exhibited significantly greater EAPC in both prevalence and YLDs compared to men throughout the study period, a disparity strongly associated with the higher prevalence of osteoporosis among elderly women.^[13–16]^

Our findings highlight distinct sex-specific patterns across age strata: in the 15 to 59 years cohort, males showed elevated incidence, prevalence, and YLDs rates, likely attributable to occupational hazards and risk-taking behaviors characteristic of younger and middle-aged men.^[17–19]^ Conversely, a striking reversal occurred beyond age 60, with women demonstrating substantially higher rates across all metrics. While the postmenopausal decline in bone mineral density is a primary biological driver of this disparity, we acknowledge that differential healthcare-seeking behavior (where men may be less likely to report minor trauma or seek evaluation for shoulder pain) and potential underdiagnosis of osteoporotic fractures in older men could also contribute to the observed magnitude of this sex-based difference.^[17,20–23]^ These results underscore the critical need for enhanced osteoporosis prevention and management strategies.^[24]^ Targeted interventions to improve bone health in aging populations could substantially reduce both the incidence of osteoporotic fractures and their associated disability burden, particularly among postmenopausal women.^[25,26]^

Analysis of CSHF epidemiology across SDI strata revealed heterogeneous trends: all SDI groups showed declining trends; however, the decline in low-SDI region was the least marked. Notably, high- and high-middle SDI countries showed significantly greater fracture burdens than middle-, low-middle, and low-SDI countries. This disparity is potentially attributable to developed nations’ higher participation in risk-prone activities (such as skiing, extreme sports), accelerated population aging, elevated osteoporotic fracture susceptibility, along with their advanced diagnostic capabilities.^[27–29]^

Global epidemiological patterns of CSHF in 2021 revealed distinct regional burdens. Eastern Europe exhibited the highest incidence rates, likely influenced by harsh climates and prevalent alcohol consumption patterns. Australia showed the most elevated age-standardized prevalence and YLDs, potentially attributable to its aging demographics and high-risk outdoor lifestyle. These findings underscore the urgent need for enhanced medical infrastructure investment and targeted public health education in both regions to mitigate the growing socioeconomic impacts of fractures amidst accelerating population aging.^[30,31]^ Comparative analysis of 1990 to 2021 trends identified East Africa as achieving the most substantial incidence reduction, paralleled by South Africa’s remarkable prevalence decline, likely reflecting successful primary healthcare system strengthening and effective international aid utilization. Conversely, the Caribbean and Oceania regions displayed concerning upward trajectories, necessitating immediate implementation of comprehensive fracture prevention strategies, including community-based osteoporosis screening programs and grassroots-level diagnostic capacity building to address this emerging public health challenge.^[32,33]^

The global burden of CSHF in 2021 exhibited substantial cross-national heterogeneity, with distinct epidemiological patterns emerging across countries. India recorded the highest absolute number of incident cases, reflecting the compounded effects of its vast population base and accelerating demographic aging.^[34]^ Afghanistan had the highest age-standardized incidence rate globally, which likely stems from conflict-exacerbated risks compounded by traffic accidents, interpersonal violence, and falls.^[35,36]^ China ranking first in absolute burden primarily reflects its massive population base and rapid aging. However, its below global average age-standardized rates stem from a complex interplay of factors, including the diagnosis and treatment rates of osteoporosis, dietary habits, socioeconomic conditions, and lifestyle.^[37–40]^ Most notably, the Syrian Arab Republic exhibited the world’s most rapid escalation in incidence, prevalence, and YLD rates. The escalating trauma burden in conflict-affected regions, exemplified by the Syrian Arab Republic, underscores the devastating public health impact of political instability and highlights an urgent need for coordinated international interventions.^[41]^

Based on the global trend projection of CSHF, fracture incidence is expected to stabilize over the next 15 years, potentially due to the stabilization of the world’s total population. By simultaneously considering the effects of age, period, and cohort, the BAPC model can more accurately predict future trends in disease burden, and the use of a Bayesian framework enhances the model’s robustness and reliability. Projections indicated that the prevalence number will rise to 7207,046 cases (95% UI: 4,654,646–9,759,445) by 2036, while the prevalence rates maintain relative stability during the 2022 to 2036 period. Similarly, the forecast shows a continued increase in total YLDs from 2022 to 2036, with the age-standardized rate remaining consistently decrease throughout this timeframe. The result suggests that while absolute numbers may grow with population changes, the relative burden of these fractures remains decrease.^[42]^

The SII (95% CI) analysis revealed a progressive reduction in disparities for incidence, prevalence, and YLDs between 1990 and 2021, underscoring the need for low- and middle-income countries to prioritize fracture prevention and treatment strategies alongside economic development initiatives. Although the SII was consistently positive (higher rates at the high-SDI end), the ConI remained negative from 1990 to 2021, indicating that, across the entire SDI distribution, the fracture burden was relatively more concentrated among lower-SDI populations. This pattern suggests a nonlinear and heterogeneous SDI–burden relationship rather than a simple monotonic gradient.

Our findings should be interpreted in light of the distinct clinical phenotypes of CSHF. Proximal humerus fractures are strongly linked to population aging, osteoporosis, and low-energy falls,^[3]^ which is consistent with the disproportionately higher burden observed among older women,^[43]^ particularly in high-SDI regions where longevity and fall risk in older adults are prominent. Accordingly, prevention strategies for humerus fractures should prioritize fall-risk assessment and mitigation, routine osteoporosis evaluation and treatment, and structured secondary fracture prevention after an index fracture to reduce recurrent events and disability.^[44]^ In contrast, scapular fractures are relatively uncommon and often indicate high-energy trauma and polytrauma.^[45]^ Therefore, prevention and care priorities include injury prevention policies (such as road safety) and strengthening acute trauma systems for timely diagnosis and management. Clavicle fractures span both high-energy injuries in younger individuals and fragility-related fractures in older adults. Thus, strategies should integrate both trauma prevention (for younger populations) and fall/fragility prevention (for older adults), while emphasizing appropriate treatment selection and rehabilitation to restore shoulder function. Beyond incidence, the sustained burden reflected by prevalence and YLDs underscores the importance of CSHF-specific rehabilitation pathways, including early mobilization when clinically appropriate, access to physiotherapy, and follow-up models focused on functional recovery to mitigate long-term disability.

## 5. Limitation

Utilizing the refined GBD 2021 methodology, we conducted a comprehensive evaluation of global CSHF epidemiology, incorporating age–sex–region stratification and 15-year incidence projections. Several methodological limitations warrant consideration: regional disparities in data quality particularly affect low- and middle-income countries where underreporting occurs due to limited healthcare access and incomplete hospitalization records; case identification relied on ICD-10 with potential coding errors and miscoding and evolving diagnostic criteria affecting temporal trend validity; fracture classification may underestimate burden when multiple fractures coexist; the definition of traumatic fractures excludes pathological fractures (fractures caused by osteoporosis or tumors, etc), which to some extent underestimates the burden of CSHF, especially in elderly women and high-SDI regions; and mild or untreated humerus/clavicle fractures likely contribute to systematic underestimation; BAPC projections do not account for emergent technologies, changing risk factors and healthcare infrastructure shifts. These long-standing data heterogeneity challenges necessitate cautious interpretation, though our approach represents the most credible available pathway for assessing shoulder fracture impacts. Future investigations will expand to examine the complete spectrum of fracture-related global burden and its implications for healthcare systems worldwide.

## Acknowledgments

We would like to thank the Institute for Health Metrics and Evaluation and the Global Burden of Disease study collaborations.

## Author contributions

**Conceptualization:** Xiaoyang Sun, Teng Yao, Fengzhen Wang.

**Data curation:** Fengzhen Wang.

**Funding acquisition:** Xiaoyang Sun.

**Validation:** Fengzhen Wang.

**Writing – original draft:** Xiaoyang Sun, Teng Yao, Fengzhen Wang.

**Writing – review & editing:** Xiaoyang Sun, Teng Yao, Fengzhen Wang.














