# Supplementary material for: Global, regional, and national burden of clavicle, scapula, or humerus fracture in 204 countries and territories, 1990 to 2021: A systematic analysis from the Global Burden of Disease Study 2021
Source: Medicine (Baltimore). 2026 May 22;105(21):e48862. doi: 10.1097/MD.0000000000048862 (PMC13201055; doi:10.1097/MD.0000000000048862)

**Supplementary figure 1.** The Number and Age-Standardized Rate of the Global Burden of Fracture of clavicle, scapula, or humerus. (A) Incidence. (B) Prevalence. (C) YLDs. YLDs Years Lived with Disability.


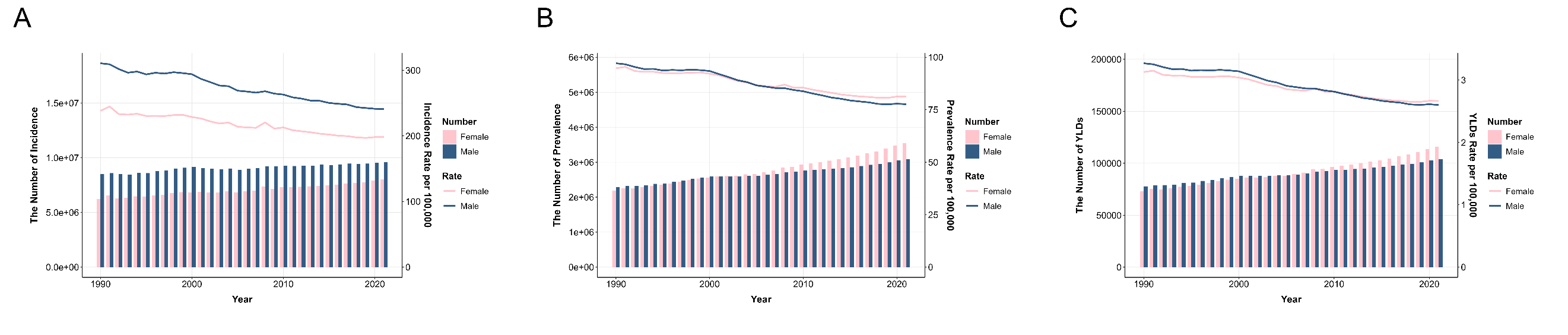

Supplement: Supplementary file 1 [file medi-105-e48862-s001.docx]
