# Supplementary material for: Global, regional, and national burden of clavicle, scapula, or humerus fracture in 204 countries and territories, 1990 to 2021: A systematic analysis from the Global Burden of Disease Study 2021
Source: Medicine (Baltimore). 2026 May 22;105(21):e48862. doi: 10.1097/MD.0000000000048862 (PMC13201055; doi:10.1097/MD.0000000000048862)

**Supplementary** **figure 2.** Age-Standardized Rates of Incidence, Prevalence, and YLDs Due to Fracture of clavicle, scapula, or humerus, by SDI Quintiles for Both Sexes, 1990–2021. (A) Incidence. (B) Prevalence. (C) YLDs. YLDs Years Lived with Disability; SDI: Socio-Demographic Index.


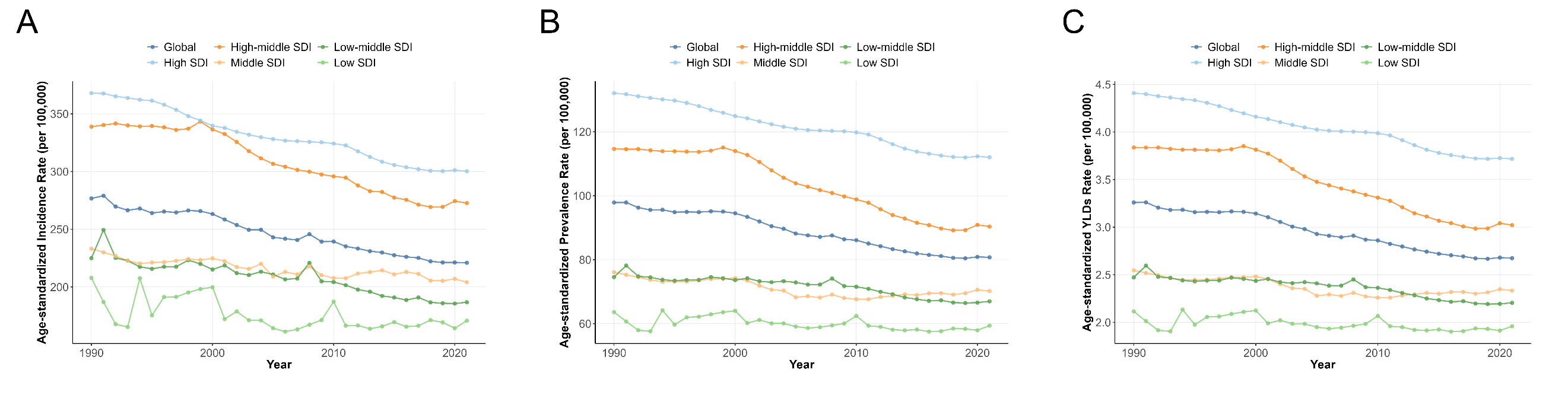

Supplement: Supplementary file 2 [file medi-105-e48862-s002.docx]
