# Supplementary material for: Global, regional, and national burden of clavicle, scapula, or humerus fracture in 204 countries and territories, 1990 to 2021: A systematic analysis from the Global Burden of Disease Study 2021
Source: Medicine (Baltimore). 2026 May 22;105(21):e48862. doi: 10.1097/MD.0000000000048862 (PMC13201055; doi:10.1097/MD.0000000000048862)
Supplement: Supplementary file 3 [file medi-105-e48862-s003.docx]

**Supplementary table 1.** **Incidence, prevalence and YLDs of Fracture of clavicle, scapula, or humerus and their EAPC among 204 countries and territories in 2021**

|  | Incidence | | | Prevalence | | | YLDs | | |
| --- | --- | --- | --- | --- | --- | --- | --- | --- | --- |
|  | Number (95% UI) | ASR per 100,000 (95% UI) | EAPC (95% CI) | Number (95% UI) | ASR per 100,000 (95% UI) | EAPC (95% CI) | Number (95% UI) | ASR per 100,000 (95% UI) | EAPC (95% CI) |
| Afghanistan | 226727 (131813,403857) | 617.59 (365.59,1059.72) | 1.43 (0.55, 2.33) | 47226 (28344,76189) | 174.38 (105.59,304.11) | 0.16 (-0.18, 0.5) | 1606 (870,2960) | 5.78 (3.16,10.32) | 0.19 (-0.18, 0.55) |
| Albania | 11279 (8549,14755) | 462.68 (349.05,607.16) | -0.95 (-1.28, -0.63) | 3551 (3031,4243) | 119.56 (98.82,147.01) | -0.8 (-1.02, -0.57) | 120 (71,189) | 4.06 (2.41,6.49) | -0.8 (-1.04, -0.57) |
| Algeria | 95895 (76690,119555) | 216.98 (173.58,270.62) | -1.46 (-1.64, -1.29) | 27757 (23445,32481) | 66.42 (56.56,77.13) | -1.3 (-1.4, -1.2) | 940 (565,1455) | 2.23 (1.35,3.45) | -1.32 (-1.42, -1.22) |
| American Samoa | 70 (55,90) | 143.88 (113.77,183.73) | -0.06 (-0.71, 0.59) | 22 (19,26) | 47.62 (40.88,55.35) | 0.1 (-0.27, 0.46) | 1 (0,1) | 1.58 (0.98,2.42) | 0.06 (-0.31, 0.44) |
| Andorra | 457 (320,630) | 478.63 (331.76,656.03) | 0.08 (0.01, 0.15) | 256 (219,298) | 194.84 (165.19,231.16) | 0.24 (0.18, 0.3) | 8 (5,13) | 6.43 (3.8,9.66) | 0.22 (0.16, 0.27) |
| Angola | 37766 (30752,46102) | 117.7 (96.18,144.38) | -2.88 (-3.93, -1.83) | 10618 (8531,13480) | 49.81 (40.78,64.58) | -1.48 (-1.98, -0.97) | 358 (223,551) | 1.64 (1.06,2.45) | -1.54 (-2.07, -1.02) |
| Antigua and Barbuda | 180 (133,250) | 208.98 (155.43,292.11) | 0 (-0.31, 0.3) | 52 (44,65) | 56.11 (46.23,70.68) | 0.03 (-0.18, 0.24) | 2 (1,3) | 1.9 (1.11,3.07) | 0.02 (-0.19, 0.23) |
| Argentina | 120172 (91949,156725) | 267.22 (203.7,350.64) | -0.17 (-0.35, 0.02) | 43461 (37249,50689) | 87.72 (74.3,103.81) | -0.22 (-0.36, -0.08) | 1455 (870,2296) | 2.95 (1.75,4.65) | -0.22 (-0.36, -0.07) |
| Armenia | 6176 (4897,7742) | 221.43 (175.24,277.65) | -1.36 (-2.24, -0.48) | 2149 (1847,2504) | 65.19 (54.29,77.03) | -1.73 (-2.37, -1.09) | 72 (45,109) | 2.2 (1.38,3.34) | -1.71 (-2.36, -1.06) |
| Australia | 111174 (81664,151137) | 427.46 (313.88,595.42) | -0.24 (-0.34, -0.14) | 52259 (44636,60384) | 149.12 (124.72,176.19) | -0.02 (-0.12, 0.07) | 1706 (1023,2586) | 4.96 (2.93,7.62) | -0.05 (-0.15, 0.04) |
| Austria | 35002 (24627,48173) | 371.07 (262.06,510.43) | -0.89 (-0.97, -0.82) | 19247 (16422,22329) | 139.22 (117.49,165.65) | -0.78 (-0.85, -0.7) | 627 (376,942) | 4.63 (2.75,7.05) | -0.79 (-0.86, -0.72) |
| Azerbaijan | 22373 (17653,28315) | 217.27 (170.77,277.32) | -0.84 (-1.47, -0.21) | 6359 (5340,7519) | 59.74 (50.09,71.1) | -0.85 (-1.24, -0.47) | 216 (132,337) | 2.03 (1.23,3.18) | -0.86 (-1.25, -0.46) |
| Bahamas | 647 (526,806) | 168.8 (136.52,211.74) | 0.36 (-0.01, 0.73) | 208 (180,241) | 52.57 (45.4,61.32) | 0.21 (0.01, 0.41) | 7 (4,11) | 1.77 (1.08,2.74) | 0.21 (0.01, 0.41) |
| Bahrain | 2951 (2339,3729) | 187.15 (148.44,237.28) | -0.47 (-0.62, -0.32) | 780 (650,927) | 52.86 (44.35,62.61) | -0.77 (-0.95, -0.59) | 27 (16,42) | 1.78 (1.05,2.78) | -0.77 (-0.95, -0.6) |
| Bangladesh | 183466 (142219,239625) | 108.23 (83.95,141.49) | -0.5 (-1.34, 0.34) | 51091 (42032,60547) | 32.16 (26.83,37.95) | -0.42 (-0.94, 0.1) | 1731 (1012,2734) | 1.08 (0.64,1.7) | -0.42 (-0.95, 0.12) |
| Barbados | 425 (337,540) | 153.72 (120.57,198.74) | -0.1 (-0.25, 0.06) | 163 (143,186) | 44.95 (38.24,53.34) | -0.01 (-0.15, 0.12) | 5 (3,8) | 1.51 (0.89,2.39) | -0.03 (-0.17, 0.11) |
| Belarus | 43700 (33719,57234) | 486.78 (376.16,643.52) | -0.27 (-0.56, 0.02) | 16222 (13993,18851) | 140.2 (117.19,167.68) | -0.27 (-0.59, 0.05) | 542 (325,845) | 4.73 (2.77,7.37) | -0.28 (-0.6, 0.04) |
| Belgium | 55600 (38584,77057) | 428.96 (298.06,592.52) | -0.07 (-0.41, 0.28) | 32368 (27878,37309) | 172.35 (146.46,202.95) | 0.16 (-0.11, 0.44) | 1048 (640,1569) | 5.71 (3.41,8.75) | 0.13 (-0.14, 0.41) |
| Belize | 924 (738,1165) | 205.2 (164.67,259.01) | 0.02 (-0.16, 0.2) | 240 (199,293) | 60.19 (50.97,71.73) | 0.16 (0.01, 0.31) | 8 (5,13) | 2.04 (1.23,3.23) | 0.15 (0, 0.29) |
| Benin | 15799 (12880,19346) | 117.72 (95.93,144.79) | -0.19 (-0.25, -0.14) | 3669 (3027,4433) | 38.96 (33.86,45.15) | -0.15 (-0.2, -0.1) | 125 (74,197) | 1.3 (0.79,1.98) | -0.14 (-0.2, -0.09) |
| Bermuda | 97 (76,124) | 168.41 (129.85,221.27) | -0.07 (-0.19, 0.04) | 41 (36,47) | 47.36 (39.69,56.72) | -0.2 (-0.29, -0.1) | 1 (1,2) | 1.6 (0.96,2.5) | -0.21 (-0.3, -0.12) |
| Bhutan | 1348 (1058,1742) | 182.5 (142.73,234.93) | 0.53 (0.19, 0.87) | 418 (350,494) | 63.01 (53.39,73.48) | 0.91 (0.7, 1.11) | 14 (8,22) | 2.09 (1.24,3.21) | 0.87 (0.66, 1.07) |
| Bolivia (Plurinational State of) | 22700 (18275,28593) | 191.12 (154.17,239.43) | -0.58 (-0.61, -0.55) | 6216 (5237,7364) | 58.14 (49.67,68.12) | -0.51 (-0.54, -0.49) | 210 (124,331) | 1.95 (1.17,3.03) | -0.53 (-0.56, -0.5) |
| Bosnia and Herzegovina | 11710 (8842,15190) | 400.55 (300.88,527.09) | -2.06 (-2.91, -1.2) | 4636 (3964,5435) | 116.19 (95.7,140.18) | -1.36 (-1.86, -0.87) | 154 (95,234) | 3.91 (2.36,6.18) | -1.4 (-1.91, -0.89) |
| Botswana | 3440 (2819,4202) | 140.42 (115.41,171.29) | 0.07 (-0.1, 0.24) | 961 (819,1140) | 46.06 (40.07,53.31) | 0.15 (-0.04, 0.34) | 33 (19,51) | 1.54 (0.91,2.36) | 0.13 (-0.05, 0.32) |
| Brazil | 651263 (514444,838821) | 294.16 (231.63,381.21) | -0.79 (-0.95, -0.64) | 216454 (184663,252420) | 91.92 (77.78,107.86) | -0.78 (-0.9, -0.66) | 7218 (4333,11099) | 3.07 (1.83,4.72) | -0.79 (-0.91, -0.67) |
| Brunei Darussalam | 1308 (1013,1705) | 283.91 (218.05,369.38) | -0.74 (-0.78, -0.7) | 431 (368,507) | 102.86 (89.02,119.49) | -0.74 (-0.78, -0.7) | 15 (9,23) | 3.44 (2.05,5.3) | -0.75 (-0.79, -0.71) |
| Bulgaria | 28985 (22168,37481) | 496.44 (380.89,643.5) | -0.86 (-0.88, -0.83) | 11065 (9623,12796) | 132.98 (110.04,161.77) | -0.8 (-0.83, -0.78) | 369 (221,576) | 4.51 (2.63,7.18) | -0.8 (-0.83, -0.78) |
| Burkina Faso | 34040 (26815,43418) | 145.87 (116.44,181.59) | 0.36 (0.14, 0.57) | 7657 (6139,9340) | 46 (39.36,53.23) | 0.17 (0.05, 0.28) | 263 (154,420) | 1.54 (0.92,2.37) | 0.19 (0.07, 0.31) |
| Burundi | 14922 (12137,18370) | 112.14 (92.1,137.39) | -4.79 (-7.27, -2.24) | 6194 (4009,10370) | 66.25 (43.63,111.14) | -0.7 (-2.23, 0.85) | 205 (122,345) | 2.16 (1.32,3.7) | -0.87 (-2.44, 0.71) |
| Cabo Verde | 705 (569,890) | 127.09 (102.71,161.17) | 0.02 (-0.03, 0.06) | 212 (182,250) | 41.23 (35.66,48.35) | 0.14 (0.1, 0.17) | 7 (4,11) | 1.38 (0.83,2.12) | 0.12 (0.08, 0.15) |
| Cambodia | 31387 (24535,40649) | 188.9 (146.31,246.8) | -0.33 (-0.61, -0.05) | 10730 (8681,14426) | 76.45 (62.6,101.93) | -0.19 (-0.3, -0.09) | 358 (222,568) | 2.52 (1.57,3.92) | -0.22 (-0.33, -0.1) |
| Cameroon | 40358 (32519,49598) | 127.77 (102.3,157.35) | 0.5 (0.36, 0.64) | 9450 (7770,11396) | 42.08 (36.45,48.4) | 0.23 (0.18, 0.29) | 323 (194,511) | 1.4 (0.85,2.16) | 0.25 (0.19, 0.31) |
| Canada | 112612 (81758,152442) | 239.46 (179.36,312.93) | -0.4 (-0.45, -0.35) | 64313 (54741,74427) | 102.88 (88.6,120.05) | -0.06 (-0.13, 0.02) | 2083 (1260,3134) | 3.39 (2.03,5.18) | -0.1 (-0.17, -0.03) |
| Central African Republic | 13106 (9651,18559) | 214.96 (163.11,292.77) | 1.69 (0.9, 2.48) | 2858 (2175,3794) | 61.19 (49.87,76.63) | 1.04 (0.64, 1.45) | 98 (57,164) | 2.05 (1.23,3.28) | 1.08 (0.66, 1.51) |
| Chad | 25461 (19939,32250) | 136.09 (109.35,169.39) | -0.44 (-0.98, 0.1) | 5612 (4356,7041) | 44.89 (37.72,53.79) | -0.18 (-0.43, 0.08) | 192 (116,307) | 1.5 (0.95,2.29) | -0.18 (-0.45, 0.08) |
| Chile | 62853 (47654,84197) | 342.3 (255.95,465.56) | 0.49 (0.36, 0.63) | 24983 (21452,29085) | 115.5 (97.21,138.04) | 0.41 (0.3, 0.53) | 829 (501,1273) | 3.86 (2.31,5.96) | 0.4 (0.29, 0.52) |
| China | 2939445 (2251270,3868896) | 199.8 (154.61,262.59) | -0.19 (-0.53, 0.15) | 1211293 (1042466,1389637) | 70.17 (59.52,81.74) | -0.17 (-0.49, 0.14) | 40235 (24471,61196) | 2.34 (1.4,3.6) | -0.19 (-0.5, 0.12) |
| Colombia | 105149 (84492,134769) | 222.46 (178.96,285.58) | -1.57 (-1.68, -1.46) | 33636 (28809,39410) | 65.88 (55.66,77.68) | -1.49 (-1.55, -1.43) | 1131 (696,1702) | 2.22 (1.35,3.36) | -1.49 (-1.56, -1.43) |
| Comoros | 782 (638,970) | 107.39 (87.86,132.66) | -0.14 (-0.48, 0.2) | 223 (189,262) | 36.23 (31.49,41.85) | -0.2 (-0.36, -0.03) | 8 (5,12) | 1.21 (0.75,1.86) | -0.2 (-0.37, -0.03) |
| Congo | 5650 (4602,6873) | 108.4 (88.26,131.77) | -1.89 (-3.4, -0.35) | 1784 (1449,2237) | 43.6 (36.64,53.14) | -0.83 (-1.76, 0.12) | 60 (38,90) | 1.44 (0.92,2.14) | -0.88 (-1.85, 0.09) |
| Cook Islands | 25 (20,33) | 145.03 (111.18,190.4) | -0.23 (-0.84, 0.37) | 10 (9,11) | 47.88 (40.72,55.63) | -0.17 (-0.5, 0.16) | 0 (0,0) | 1.59 (0.95,2.43) | -0.18 (-0.52, 0.15) |
| Costa Rica | 10614 (8264,13934) | 231.95 (180.98,303.04) | -0.32 (-0.38, -0.27) | 3600 (3080,4185) | 71.16 (60.13,83.99) | -0.38 (-0.41, -0.35) | 120 (71,185) | 2.38 (1.4,3.72) | -0.38 (-0.41, -0.35) |
| Croatia | 22046 (16678,29466) | 469.06 (359.83,617.29) | -1.38 (-1.71, -1.05) | 10315 (8775,11951) | 153.97 (128.45,181.58) | -0.78 (-0.96, -0.6) | 334 (202,503) | 5.1 (3.05,7.82) | -0.83 (-1.02, -0.65) |
| Cuba | 31819 (24714,41711) | 257.18 (201.11,331.63) | 0.19 (0.1, 0.29) | 14354 (12144,16597) | 87.3 (74.12,102.27) | 0.29 (0.22, 0.36) | 466 (282,705) | 2.89 (1.72,4.47) | 0.26 (0.19, 0.33) |
| Cyprus | 4905 (3554,6650) | 363.51 (260.9,492.69) | -0.41 (-0.57, -0.24) | 2220 (1910,2566) | 134.91 (115.09,159.34) | -0.42 (-0.56, -0.27) | 74 (44,111) | 4.5 (2.68,6.79) | -0.42 (-0.56, -0.27) |
| Czechia | 49987 (38003,64528) | 481.39 (366.46,633.65) | -1.15 (-1.33, -0.98) | 20651 (17863,23893) | 140.81 (117.56,167.63) | -1.53 (-1.66, -1.41) | 676 (404,1028) | 4.71 (2.78,7.39) | -1.49 (-1.62, -1.36) |
| Côte d'Ivoire | 31521 (25701,39126) | 117.98 (96.18,145.65) | -0.15 (-0.38, 0.08) | 7654 (6329,9179) | 39.8 (34.74,46.08) | -0.11 (-0.22, 0.01) | 261 (154,404) | 1.33 (0.81,2.04) | -0.09 (-0.22, 0.03) |
| Democratic People's Republic of Korea | 31633 (25874,38554) | 113.55 (93.22,138.69) | -0.52 (-0.59, -0.45) | 12315 (10940,13979) | 40.56 (35.76,46.2) | -0.54 (-0.58, -0.5) | 415 (250,629) | 1.37 (0.82,2.07) | -0.54 (-0.58, -0.49) |
| Democratic Republic of the Congo | 117212 (95235,143109) | 129.11 (106.16,156.63) | -0.8 (-1.69, 0.09) | 29388 (24016,35863) | 45.18 (38.55,52.63) | -0.28 (-0.76, 0.21) | 997 (607,1545) | 1.5 (0.94,2.29) | -0.29 (-0.79, 0.21) |
| Denmark | 18160 (12781,25010) | 302.47 (212.93,421.53) | -0.92 (-1.03, -0.81) | 9588 (8193,11071) | 110.87 (93.09,131.46) | -1.27 (-1.36, -1.18) | 314 (191,463) | 3.71 (2.18,5.59) | -1.22 (-1.32, -1.13) |
| Djibouti | 1346 (1101,1663) | 111.54 (90.84,137.35) | -1.17 (-1.56, -0.78) | 354 (296,421) | 37.22 (32.16,43.13) | -0.62 (-0.79, -0.46) | 12 (7,19) | 1.24 (0.76,1.94) | -0.65 (-0.82, -0.47) |
| Dominica | 103 (84,129) | 156.97 (127,198.62) | 0.42 (0.01, 0.84) | 34 (29,39) | 46.83 (39.85,55.02) | 0.29 (0.05, 0.53) | 1 (1,2) | 1.58 (0.94,2.46) | 0.28 (0.04, 0.53) |
| Dominican Republic | 21145 (17208,26300) | 188.91 (153.84,235.08) | 0.56 (0.38, 0.75) | 6062 (5186,7172) | 55.93 (48.06,65.97) | 0.59 (0.42, 0.75) | 205 (121,325) | 1.89 (1.11,2.98) | 0.57 (0.41, 0.74) |
| Ecuador | 42759 (34366,53541) | 232.15 (186.95,290.74) | -0.03 (-0.18, 0.12) | 12306 (10403,14452) | 69.81 (59.49,81.66) | 0 (-0.14, 0.15) | 415 (244,654) | 2.35 (1.39,3.68) | 0 (-0.15, 0.15) |
| Egypt | 216605 (175166,264718) | 201.85 (163.4,245.83) | -0.78 (-0.87, -0.69) | 56098 (46850,67166) | 61.57 (52.76,72.34) | -0.7 (-0.77, -0.63) | 1907 (1151,2941) | 2.07 (1.27,3.13) | -0.71 (-0.78, -0.64) |
| El Salvador | 15839 (12631,20013) | 242.68 (194.02,306.84) | -0.79 (-1.12, -0.45) | 5697 (4726,6958) | 89.07 (73.66,109.7) | -0.92 (-1.04, -0.8) | 189 (119,282) | 2.96 (1.87,4.41) | -0.93 (-1.06, -0.8) |
| Equatorial Guinea | 1526 (1226,1890) | 105.07 (84.78,128.82) | -0.65 (-0.7, -0.59) | 365 (303,437) | 36.67 (31.94,42.02) | -0.54 (-0.59, -0.5) | 12 (7,19) | 1.22 (0.72,1.85) | -0.55 (-0.59, -0.5) |
| Eritrea | 7405 (6086,9151) | 118.06 (96.76,145.91) | -2.87 (-4.59, -1.11) | 3213 (2117,5475) | 73.92 (48.21,128.59) | -1.97 (-2.55, -1.39) | 106 (63,172) | 2.38 (1.43,3.98) | -2.04 (-2.66, -1.42) |
| Estonia | 4678 (3606,6142) | 383.64 (297.71,502.27) | -1.96 (-2.07, -1.85) | 1848 (1613,2123) | 108.59 (90.8,129.61) | -2.14 (-2.27, -2.01) | 61 (36,93) | 3.67 (2.19,5.76) | -2.13 (-2.25, -2) |
| Eswatini | 2061 (1694,2575) | 168.8 (139.88,207.39) | 0.22 (0.06, 0.39) | 492 (407,596) | 50.86 (43.77,59.4) | 0.09 (-0.08, 0.27) | 17 (10,27) | 1.7 (1.02,2.66) | 0.08 (-0.1, 0.25) |
| Ethiopia | 169385 (118501,251070) | 148.71 (109.78,210.1) | -2.31 (-3.29, -1.31) | 37288 (27692,51546) | 45.09 (36.11,58.77) | -1.76 (-2.27, -1.25) | 1273 (730,2160) | 1.5 (0.91,2.43) | -1.78 (-2.32, -1.25) |
| Fiji | 990 (795,1224) | 108.18 (86.65,133.95) | -0.4 (-0.54, -0.26) | 295 (251,345) | 35.22 (30.46,40.9) | -0.33 (-0.43, -0.24) | 10 (6,16) | 1.18 (0.72,1.83) | -0.35 (-0.45, -0.25) |
| Finland | 27080 (18711,37289) | 454.47 (313.1,632.22) | -0.62 (-1.1, -0.13) | 15891 (13732,18393) | 172.6 (145.09,207.32) | -0.59 (-1.03, -0.14) | 515 (315,767) | 5.74 (3.37,8.81) | -0.59 (-1.03, -0.14) |
| France | 295852 (206979,403963) | 397.59 (279.69,543.78) | -0.57 (-0.65, -0.48) | 170486 (147364,196444) | 154.41 (131.96,181.51) | -0.49 (-0.57, -0.4) | 5516 (3348,8301) | 5.13 (3.02,7.98) | -0.48 (-0.57, -0.4) |
| Gabon | 2114 (1727,2578) | 120.59 (98.59,146.71) | -0.41 (-0.44, -0.38) | 580 (491,683) | 41.99 (36.58,48.24) | -0.4 (-0.43, -0.38) | 20 (12,31) | 1.4 (0.82,2.14) | -0.41 (-0.43, -0.38) |
| Gambia | 2386 (1912,3001) | 105.12 (84.38,130.88) | -0.04 (-0.15, 0.06) | 588 (485,708) | 37.09 (31.85,42.81) | 0.08 (0, 0.15) | 20 (12,31) | 1.23 (0.74,1.89) | 0.06 (-0.01, 0.13) |
| Georgia | 13770 (10894,17577) | 418.77 (330.71,537.17) | 0.15 (-0.22, 0.51) | 4849 (4150,5598) | 116.39 (97.09,138.54) | 0.38 (0.11, 0.64) | 162 (97,249) | 3.94 (2.33,6.07) | 0.35 (0.09, 0.62) |
| Germany | 304422 (213509,415951) | 327.58 (229.87,452.06) | -0.6 (-0.65, -0.56) | 178796 (153633,207331) | 124.12 (104.62,147.11) | -0.52 (-0.56, -0.48) | 5783 (3507,8687) | 4.12 (2.44,6.28) | -0.54 (-0.58, -0.5) |
| Ghana | 36508 (29283,45990) | 110.74 (89.01,138.49) | 0.04 (-0.04, 0.11) | 9383 (7864,11119) | 38.22 (33.03,43.97) | 0.06 (-0.01, 0.12) | 320 (187,506) | 1.28 (0.76,1.98) | 0.06 (-0.01, 0.13) |
| Greece | 27924 (20643,36767) | 304.14 (222.1,414.57) | -1.07 (-1.19, -0.95) | 15291 (13403,17387) | 104.4 (88.49,122.86) | -1.13 (-1.23, -1.03) | 503 (303,769) | 3.51 (2.03,5.41) | -1.12 (-1.22, -1.02) |
| Greenland | 200 (147,269) | 328.91 (244.31,440.14) | -0.8 (-0.9, -0.7) | 90 (77,105) | 146.3 (125.74,170.88) | -0.85 (-0.94, -0.76) | 3 (2,5) | 4.82 (2.92,7.41) | -0.84 (-0.92, -0.75) |
| Grenada | 198 (153,256) | 194.7 (150.06,251.11) | 0.19 (0.03, 0.34) | 61 (51,72) | 57.5 (47.73,68.98) | 0.07 (-0.03, 0.17) | 2 (1,3) | 1.93 (1.16,3.04) | 0.06 (-0.04, 0.16) |
| Guam | 198 (154,256) | 126.16 (98.18,161.97) | -0.2 (-0.34, -0.07) | 75 (66,86) | 41.61 (35.6,48.64) | -0.16 (-0.25, -0.07) | 3 (2,4) | 1.4 (0.84,2.14) | -0.16 (-0.25, -0.07) |
| Guatemala | 47072 (37110,60797) | 289.38 (229.1,375.03) | -0.19 (-0.31, -0.07) | 12924 (10632,15658) | 92.17 (77.31,109.08) | -0.33 (-0.37, -0.29) | 434 (267,663) | 3.07 (1.89,4.66) | -0.34 (-0.39, -0.3) |
| Guinea | 15832 (12925,19445) | 117.26 (96.36,143.44) | -0.07 (-0.39, 0.26) | 3822 (3171,4566) | 39.25 (34.2,45.21) | 0.08 (-0.08, 0.24) | 130 (76,208) | 1.31 (0.78,2.02) | 0.08 (-0.09, 0.25) |
| Guinea-Bissau | 2452 (2008,2983) | 125.11 (102.83,152.4) | -0.82 (-1.41, -0.24) | 602 (500,722) | 44.47 (39.05,50.88) | -0.49 (-0.8, -0.18) | 21 (12,33) | 1.48 (0.91,2.25) | -0.5 (-0.82, -0.18) |
| Guyana | 1728 (1417,2157) | 223.26 (183.66,278.95) | 0.22 (0.03, 0.42) | 491 (418,579) | 68.89 (59.25,80.13) | 0.18 (0.05, 0.32) | 17 (10,25) | 2.3 (1.39,3.53) | 0.18 (0.04, 0.32) |
| Haiti | 32007 (25574,40803) | 237.07 (189.3,301.02) | 0.08 (-1.32, 1.49) | 8370 (6557,10962) | 74.26 (58.69,97.12) | 0.82 (-0.13, 1.79) | 283 (169,436) | 2.48 (1.52,3.72) | 0.77 (-0.21, 1.75) |
| Honduras | 22259 (17773,28414) | 215.04 (171.57,274.57) | -0.83 (-1.37, -0.29) | 5849 (4901,7073) | 66.45 (57.37,78.17) | -0.56 (-0.89, -0.23) | 199 (118,304) | 2.23 (1.34,3.36) | -0.58 (-0.92, -0.25) |
| Hungary | 43299 (32738,56295) | 444.13 (338.52,587.38) | -1.42 (-1.54, -1.3) | 18843 (16262,21734) | 135 (113.42,160.01) | -1.86 (-1.99, -1.74) | 617 (370,945) | 4.52 (2.63,7.05) | -1.81 (-1.93, -1.68) |
| Iceland | 1124 (784,1555) | 324.61 (224.69,452.13) | -0.51 (-0.6, -0.42) | 534 (460,618) | 117.47 (98.26,139.4) | -0.53 (-0.59, -0.46) | 18 (11,26) | 3.94 (2.3,6.03) | -0.52 (-0.59, -0.46) |
| India | 3034155 (2318236,4010419) | 219.22 (166.36,293.24) | -0.56 (-0.63, -0.5) | 1029574 (863670,1210340) | 85.05 (71.94,99.34) | -0.33 (-0.37, -0.28) | 33980 (20560,52299) | 2.77 (1.68,4.2) | -0.34 (-0.38, -0.29) |
| Indonesia | 381977 (305420,480431) | 140.8 (112.04,176.75) | -1.04 (-1.19, -0.89) | 118245 (101581,138130) | 48.28 (41.32,55.94) | -1.06 (-1.16, -0.97) | 3985 (2420,6094) | 1.6 (0.99,2.42) | -1.07 (-1.17, -0.97) |
| Iran (Islamic Republic of) | 193870 (157976,241622) | 227.92 (185.75,282.29) | -1.42 (-1.55, -1.28) | 61727 (52294,71366) | 72.2 (61.4,83.72) | -1.48 (-1.59, -1.37) | 2073 (1272,3121) | 2.42 (1.48,3.64) | -1.49 (-1.6, -1.38) |
| Iraq | 118574 (95813,147920) | 273.96 (221.54,340.64) | 0.34 (-0.72, 1.41) | 38688 (29152,54080) | 106.46 (79.76,151.29) | -0.05 (-0.56, 0.46) | 1292 (830,1971) | 3.51 (2.29,5.3) | -0.06 (-0.59, 0.48) |
| Ireland | 15371 (10660,21172) | 320.34 (222.42,445.95) | -0.34 (-0.53, -0.15) | 7120 (6154,8264) | 115.64 (97.85,135.82) | -0.34 (-0.53, -0.15) | 235 (143,360) | 3.87 (2.28,5.98) | -0.34 (-0.54, -0.15) |
| Israel | 26664 (19287,36205) | 280.19 (202.85,379.43) | -0.32 (-0.62, -0.02) | 10186 (8750,11948) | 96.8 (82.55,114.85) | -0.23 (-0.44, -0.01) | 340 (209,520) | 3.25 (1.99,4.99) | -0.23 (-0.45, -0.01) |
| Italy | 220166 (154587,296740) | 367.06 (257.98,505.79) | -1.07 (-1.13, -1) | 130396 (112220,150793) | 131.81 (110.6,158.11) | -1.18 (-1.23, -1.14) | 4230 (2565,6296) | 4.4 (2.57,6.68) | -1.16 (-1.21, -1.11) |
| Jamaica | 5151 (4005,6687) | 184.96 (144.23,237.01) | -0.09 (-0.23, 0.05) | 1478 (1246,1751) | 49.89 (41.56,59.78) | -0.05 (-0.17, 0.08) | 50 (30,80) | 1.69 (1.01,2.71) | -0.06 (-0.19, 0.06) |
| Japan | 270034 (197409,362621) | 208.19 (155.2,285.52) | -1.27 (-1.42, -1.11) | 169929 (146884,193882) | 74.18 (63.07,87.55) | -1.24 (-1.4, -1.08) | 5540 (3329,8362) | 2.5 (1.48,3.85) | -1.24 (-1.4, -1.08) |
| Jordan | 22315 (17581,28234) | 172.03 (135.67,217.27) | -0.77 (-0.83, -0.71) | 5525 (4538,6679) | 49.16 (41.53,58.47) | -0.87 (-0.92, -0.81) | 189 (112,294) | 1.66 (0.98,2.57) | -0.87 (-0.93, -0.82) |
| Kazakhstan | 62525 (49980,79545) | 331.19 (264.36,423.26) | -0.42 (-0.54, -0.3) | 17617 (14894,20695) | 93.81 (79.6,110.32) | -0.38 (-0.5, -0.25) | 596 (354,938) | 3.17 (1.89,4.96) | -0.39 (-0.52, -0.26) |
| Kenya | 51401 (41835,63756) | 112.06 (90.7,140.08) | -0.25 (-0.46, -0.03) | 13203 (11040,15725) | 39.03 (33.81,44.88) | -0.09 (-0.25, 0.07) | 449 (269,698) | 1.29 (0.8,2) | -0.1 (-0.26, 0.06) |
| Kiribati | 108 (88,133) | 87.37 (71.31,107.61) | -0.81 (-1.17, -0.44) | 31 (26,36) | 30.7 (26.98,35.06) | -0.57 (-0.76, -0.39) | 1 (1,2) | 1.03 (0.63,1.58) | -0.57 (-0.77, -0.38) |
| Kuwait | 10653 (8281,13760) | 220.76 (171.95,283.11) | -1.48 (-2.2, -0.76) | 3001 (2465,3590) | 63.39 (52.75,75.53) | -1.23 (-1.64, -0.82) | 102 (61,154) | 2.14 (1.28,3.2) | -1.25 (-1.67, -0.83) |
| Kyrgyzstan | 16392 (13280,20654) | 230.92 (187.78,289.7) | -1.5 (-1.62, -1.39) | 4119 (3436,4945) | 64.37 (54.41,76.12) | -1.48 (-1.55, -1.4) | 141 (83,221) | 2.19 (1.29,3.37) | -1.48 (-1.55, -1.4) |
| Lao People's Democratic Republic | 9973 (8157,12257) | 134.06 (109.8,165.5) | -0.74 (-0.92, -0.56) | 2726 (2307,3214) | 44.11 (38.48,50.75) | -0.7 (-0.79, -0.61) | 93 (55,145) | 1.48 (0.88,2.27) | -0.71 (-0.8, -0.61) |
| Latvia | 7289 (5605,9347) | 410.72 (320.47,528.15) | -2.23 (-2.41, -2.05) | 3021 (2649,3463) | 119.2 (101.04,140.69) | -2.42 (-2.63, -2.22) | 100 (59,154) | 4.02 (2.38,6.22) | -2.4 (-2.61, -2.2) |
| Lebanon | 10345 (8251,13062) | 184.93 (146.64,233.76) | -1.6 (-2.09, -1.11) | 3855 (3015,5561) | 66.15 (51.56,96.32) | -1.79 (-2.06, -1.52) | 127 (79,200) | 2.19 (1.35,3.47) | -1.8 (-2.07, -1.52) |
| Lesotho | 3065 (2512,3760) | 159.54 (130.67,195.2) | 0.86 (0.67, 1.05) | 847 (722,1001) | 54.5 (47.87,62.95) | 1.03 (0.85, 1.21) | 29 (17,44) | 1.81 (1.09,2.77) | 0.99 (0.81, 1.17) |
| Liberia | 5280 (4253,6633) | 100.05 (80.46,124.94) | -4.45 (-6.03, -2.85) | 1672 (1276,2277) | 43.44 (34.4,57.43) | -1.95 (-2.81, -1.09) | 56 (35,86) | 1.43 (0.92,2.15) | -2.07 (-2.96, -1.17) |
| Libya | 17831 (14575,21840) | 268.64 (217.69,334.01) | 1.72 (0.82, 2.63) | 5623 (4740,6657) | 87.12 (73.81,102.8) | 1.26 (0.72, 1.81) | 189 (118,285) | 2.91 (1.82,4.38) | 1.25 (0.69, 1.81) |
| Lithuania | 11505 (8849,15015) | 424.85 (330.23,549.35) | -1.48 (-1.67, -1.29) | 5026 (4425,5740) | 129.12 (109.69,151.68) | -1.42 (-1.62, -1.23) | 166 (101,257) | 4.34 (2.57,6.81) | -1.43 (-1.62, -1.24) |
| Luxembourg | 2490 (1755,3378) | 380.26 (266.68,521.89) | -0.77 (-0.81, -0.72) | 1241 (1066,1432) | 142.72 (120.25,170.03) | -0.67 (-0.72, -0.63) | 41 (25,61) | 4.76 (2.81,7.3) | -0.69 (-0.73, -0.65) |
| Madagascar | 27214 (22001,34049) | 94.27 (77.17,117.83) | -0.58 (-0.65, -0.52) | 6225 (5114,7606) | 29.92 (25.92,34.55) | -0.52 (-0.57, -0.47) | 214 (122,345) | 1 (0.6,1.57) | -0.52 (-0.57, -0.47) |
| Malawi | 18919 (15210,23633) | 98.4 (79.09,123) | -0.37 (-0.43, -0.3) | 4408 (3626,5339) | 33.32 (28.74,38.72) | -0.22 (-0.28, -0.16) | 151 (87,241) | 1.11 (0.66,1.7) | -0.22 (-0.28, -0.16) |
| Malaysia | 47247 (38150,58751) | 145.73 (117.87,180.98) | -0.14 (-0.17, -0.12) | 14428 (12454,16831) | 47.72 (41.45,55.13) | -0.11 (-0.13, -0.09) | 486 (286,760) | 1.59 (0.95,2.47) | -0.12 (-0.14, -0.1) |
| Maldives | 799 (623,1035) | 151.03 (117.81,196.31) | -0.36 (-0.82, 0.1) | 221 (184,263) | 47.38 (40.28,55.85) | -0.21 (-0.47, 0.04) | 8 (4,12) | 1.59 (0.95,2.45) | -0.24 (-0.5, 0.02) |
| Mali | 33861 (26722,42245) | 137.01 (109.58,168.36) | -0.84 (-1.87, 0.21) | 7937 (6307,9807) | 47.32 (39.9,55.87) | -0.23 (-0.79, 0.33) | 270 (166,420) | 1.57 (1,2.35) | -0.26 (-0.84, 0.33) |
| Malta | 1463 (1024,2043) | 346.46 (241.72,485.63) | -0.1 (-0.31, 0.11) | 805 (694,929) | 123.75 (103.61,147.25) | -0.22 (-0.4, -0.05) | 26 (16,40) | 4.15 (2.37,6.36) | -0.22 (-0.39, -0.04) |
| Marshall Islands | 77 (62,96) | 136.38 (110.29,169.71) | -0.11 (-0.17, -0.04) | 22 (19,26) | 46.85 (40.7,54.01) | -0.1 (-0.16, -0.03) | 1 (0,1) | 1.56 (0.93,2.37) | -0.11 (-0.17, -0.04) |
| Mauritania | 4823 (3913,5939) | 112.72 (91.61,138.07) | -0.62 (-0.68, -0.56) | 1241 (1038,1464) | 38.73 (33.47,44.45) | -0.66 (-0.7, -0.62) | 42 (25,66) | 1.29 (0.78,1.97) | -0.66 (-0.7, -0.62) |
| Mauritius | 1582 (1238,2022) | 124.77 (98.03,159.76) | 0.15 (0.05, 0.25) | 566 (489,656) | 37.88 (32.08,44.77) | 0.19 (0.1, 0.29) | 19 (12,29) | 1.27 (0.77,1.95) | 0.18 (0.09, 0.27) |
| Mexico | 332311 (260383,430547) | 257.43 (201.46,334.87) | -0.41 (-0.85, 0.04) | 102966 (87282,120474) | 80.15 (67.96,93.97) | -0.6 (-0.96, -0.24) | 3456 (2064,5306) | 2.68 (1.6,4.13) | -0.59 (-0.95, -0.22) |
| Micronesia (Federated States of) | 154 (125,189) | 149.81 (121.11,185.8) | 0.06 (-0.33, 0.44) | 46 (39,54) | 52.57 (45.66,60.66) | 0.14 (-0.05, 0.32) | 2 (1,2) | 1.76 (1.05,2.64) | 0.12 (-0.07, 0.32) |
| Monaco | 106 (75,144) | 299.95 (211.43,415.62) | 0 (-0.13, 0.12) | 63 (55,73) | 105.34 (88.13,126.08) | 0 (-0.13, 0.12) | 2 (1,3) | 3.54 (2.07,5.48) | -0.01 (-0.13, 0.12) |
| Mongolia | 12087 (9650,15154) | 354.47 (282.41,444.54) | 0.51 (0.4, 0.61) | 3156 (2627,3748) | 101 (85.21,118.73) | 0.4 (0.32, 0.47) | 108 (64,167) | 3.42 (2.04,5.31) | 0.4 (0.32, 0.48) |
| Montenegro | 2623 (2012,3412) | 448.64 (343.98,588.34) | -0.56 (-0.63, -0.49) | 887 (759,1035) | 124.8 (104.02,149.52) | -0.55 (-0.63, -0.48) | 30 (18,46) | 4.21 (2.5,6.66) | -0.56 (-0.63, -0.48) |
| Morocco | 89066 (72051,109685) | 238.28 (192.72,293.34) | -0.83 (-0.92, -0.74) | 26073 (22397,30845) | 72.52 (62.5,85.38) | -0.68 (-0.76, -0.59) | 877 (525,1350) | 2.43 (1.45,3.73) | -0.71 (-0.8, -0.63) |
| Mozambique | 38925 (31215,48039) | 125.45 (102.31,153.46) | -0.29 (-0.66, 0.08) | 9858 (7791,12353) | 48.86 (39.85,63.49) | -0.45 (-0.58, -0.33) | 334 (207,526) | 1.61 (1.03,2.46) | -0.46 (-0.59, -0.32) |
| Myanmar | 140618 (107365,179810) | 246.31 (189.35,313.58) | -0.09 (-1.06, 0.89) | 37645 (30939,44585) | 71.19 (59.73,83.82) | -0.04 (-0.6, 0.53) | 1271 (761,2019) | 2.38 (1.44,3.76) | -0.06 (-0.64, 0.53) |
| Namibia | 3174 (2615,3852) | 128.58 (106.19,155.88) | -0.19 (-0.38, 0) | 897 (753,1063) | 45.06 (38.74,52.5) | -0.42 (-0.56, -0.29) | 30 (19,46) | 1.51 (0.93,2.26) | -0.43 (-0.57, -0.29) |
| Nauru | 19 (15,24) | 174.58 (140.47,217.04) | 0.16 (0.06, 0.26) | 5 (4,6) | 60.5 (52.45,69.51) | 0.21 (0.09, 0.32) | 0 (0,0) | 2.02 (1.19,3.05) | 0.18 (0.07, 0.29) |
| Nepal | 71401 (52139,96010) | 226.56 (166.27,304.83) | 0.03 (-0.64, 0.7) | 21420 (17773,25806) | 79.4 (67.4,93.43) | 0.33 (0.01, 0.66) | 717 (428,1103) | 2.63 (1.58,3.99) | 0.32 (-0.02, 0.66) |
| Netherlands | 59603 (42033,82296) | 286.05 (203.55,395.78) | 0.31 (-0.07, 0.69) | 33928 (28777,39308) | 117.29 (99.45,138.31) | 0.75 (0.25, 1.24) | 1096 (662,1639) | 3.86 (2.29,5.93) | 0.7 (0.21, 1.18) |
| New Zealand | 24260 (18073,32744) | 489.16 (364.06,662.1) | -0.51 (-0.67, -0.34) | 10351 (8861,11893) | 162.15 (135.72,191.59) | -0.52 (-0.64, -0.4) | 341 (204,514) | 5.42 (3.15,8.22) | -0.52 (-0.64, -0.39) |
| Nicaragua | 12867 (9957,16786) | 194.15 (151.26,253.44) | -0.49 (-0.82, -0.16) | 4297 (3411,5509) | 73.76 (58.79,95.02) | -0.75 (-0.89, -0.61) | 143 (91,221) | 2.43 (1.53,3.73) | -0.77 (-0.91, -0.62) |
| Niger | 34124 (26779,43046) | 132.63 (106,162.54) | 0.12 (-0.04, 0.28) | 7338 (5845,9016) | 43.15 (37.06,49.78) | 0.05 (-0.04, 0.13) | 252 (148,394) | 1.44 (0.88,2.2) | 0.05 (-0.03, 0.13) |
| Nigeria | 258224 (210884,318581) | 113.17 (92.22,141.49) | -0.19 (-0.32, -0.05) | 61752 (51053,74083) | 38.69 (33.44,44.48) | -0.13 (-0.21, -0.05) | 2104 (1252,3319) | 1.28 (0.79,1.95) | -0.13 (-0.21, -0.05) |
| Niue | 2 (2,3) | 135.82 (106.21,173.88) | -0.23 (-0.66, 0.19) | 1 (1,1) | 45.47 (39.07,52.39) | -0.18 (-0.42, 0.05) | 0 (0,0) | 1.52 (0.91,2.35) | -0.21 (-0.45, 0.03) |
| North Macedonia | 8173 (6294,10498) | 406.11 (308.62,528.33) | -0.71 (-0.83, -0.58) | 2671 (2292,3129) | 111.6 (92.58,135.04) | -0.33 (-0.47, -0.19) | 89 (53,139) | 3.76 (2.22,5.96) | -0.36 (-0.5, -0.23) |
| Northern Mariana Islands | 91 (71,116) | 191.25 (151.14,246.19) | -0.24 (-0.33, -0.16) | 30 (26,35) | 63.94 (55.28,74.83) | -0.11 (-0.15, -0.06) | 1 (1,2) | 2.13 (1.26,3.29) | -0.13 (-0.18, -0.09) |
| Norway | 19487 (13581,26968) | 315.84 (223.78,435.43) | -0.78 (-0.93, -0.62) | 10625 (8928,12537) | 125.28 (104.63,149.02) | -0.75 (-0.89, -0.62) | 343 (205,514) | 4.13 (2.45,6.29) | -0.76 (-0.9, -0.62) |
| Oman | 13634 (10938,17059) | 291.76 (233.91,364.64) | -1.06 (-1.19, -0.93) | 3566 (2963,4251) | 92.65 (79.25,107.47) | -1.18 (-1.33, -1.04) | 122 (71,189) | 3.09 (1.86,4.74) | -1.2 (-1.34, -1.05) |
| Pakistan | 266096 (216233,324589) | 110.89 (90.08,136.02) | 0.08 (-0.46, 0.63) | 70084 (58666,83017) | 36.26 (31.3,41.98) | 0.07 (-0.27, 0.41) | 2375 (1458,3612) | 1.21 (0.76,1.81) | 0.06 (-0.28, 0.41) |
| Palau | 43 (33,55) | 239.2 (185.76,310.79) | 0.26 (0.23, 0.28) | 15 (13,18) | 79.07 (66.97,92.96) | 0.12 (0.1, 0.14) | 1 (0,1) | 2.63 (1.57,4.03) | 0.12 (0.1, 0.13) |
| Palestine | 12428 (9699,15838) | 224.68 (175.76,284.14) | -1.02 (-2.64, 0.62) | 3763 (2771,5308) | 87.47 (65.32,124.99) | -0.07 (-0.89, 0.75) | 126 (81,197) | 2.89 (1.87,4.49) | -0.14 (-0.99, 0.73) |
| Panama | 8678 (6791,11355) | 203.68 (159.29,265.94) | -0.63 (-0.69, -0.57) | 2530 (2138,2983) | 58.17 (48.96,68.76) | -0.76 (-0.8, -0.72) | 85 (51,132) | 1.96 (1.18,3.05) | -0.75 (-0.8, -0.71) |
| Papua New Guinea | 17990 (14186,22446) | 180.91 (141.24,225.43) | 0.12 (-0.37, 0.62) | 4884 (4132,5818) | 67.41 (58.22,77.4) | 0.39 (0.15, 0.63) | 165 (96,255) | 2.22 (1.33,3.37) | 0.38 (0.13, 0.63) |
| Paraguay | 18773 (14884,23958) | 253.48 (200.72,322.1) | -0.25 (-0.34, -0.17) | 5045 (4183,5990) | 73.44 (61.75,86.46) | -0.13 (-0.22, -0.05) | 171 (101,265) | 2.47 (1.47,3.79) | -0.15 (-0.24, -0.06) |
| Peru | 70191 (55689,90167) | 190.56 (151.49,244.49) | -0.68 (-0.92, -0.44) | 20174 (17010,23611) | 55.92 (47.38,65.31) | -0.46 (-0.57, -0.35) | 682 (401,1054) | 1.89 (1.1,2.92) | -0.47 (-0.59, -0.36) |
| Philippines | 150209 (120852,188662) | 131.43 (105.62,165.09) | -0.77 (-1.06, -0.48) | 44668 (37780,52814) | 44.76 (38.61,52.13) | -0.74 (-0.88, -0.6) | 1508 (935,2272) | 1.49 (0.93,2.23) | -0.74 (-0.89, -0.6) |
| Poland | 163306 (123276,213520) | 430.2 (327.63,566.08) | -0.98 (-1.07, -0.89) | 64307 (55641,74273) | 126.24 (105.23,150.39) | -1.07 (-1.16, -0.97) | 2122 (1281,3304) | 4.24 (2.5,6.66) | -1.05 (-1.15, -0.96) |
| Portugal | 26994 (20110,35906) | 247.05 (181.88,331.22) | -2.01 (-2.11, -1.92) | 15673 (13650,17772) | 91.49 (78.33,106.76) | -1.74 (-1.82, -1.67) | 512 (314,777) | 3.05 (1.86,4.74) | -1.77 (-1.85, -1.69) |
| Puerto Rico | 7321 (5779,9432) | 226.87 (180.59,293.95) | 0.3 (0.14, 0.47) | 3270 (2872,3768) | 67.6 (57.51,79.17) | 0.23 (0.13, 0.33) | 107 (65,167) | 2.28 (1.36,3.56) | 0.22 (0.11, 0.32) |
| Qatar | 9187 (7204,11678) | 273.98 (216.85,348.18) | -0.37 (-0.49, -0.25) | 2242 (1808,2721) | 78.06 (65.11,92.63) | -0.49 (-0.62, -0.36) | 77 (45,125) | 2.62 (1.55,4.09) | -0.51 (-0.64, -0.38) |
| Republic of Korea | 175153 (128891,232086) | 313.79 (230.36,420.27) | -1.72 (-1.82, -1.62) | 88748 (76846,101756) | 119.81 (103.04,140.85) | -1.44 (-1.53, -1.34) | 2924 (1779,4407) | 3.99 (2.36,6.09) | -1.47 (-1.57, -1.38) |
| Republic of Moldova | 11006 (8731,14023) | 326.47 (259.09,416.19) | -1.83 (-1.92, -1.73) | 4065 (3524,4662) | 93.71 (78.85,110.77) | -1.81 (-1.88, -1.74) | 136 (80,212) | 3.18 (1.85,4.96) | -1.81 (-1.88, -1.74) |
| Romania | 81151 (62072,104147) | 467.6 (360.72,609.8) | -1.18 (-1.24, -1.12) | 30830 (26718,35753) | 129.55 (107.6,155.68) | -1.17 (-1.23, -1.11) | 1030 (634,1596) | 4.4 (2.61,6.96) | -1.17 (-1.23, -1.1) |
| Russian Federation | 644655 (508393,835808) | 463.35 (369.08,601.06) | -1.15 (-1.49, -0.8) | 238590 (207790,273281) | 135.95 (115.5,159.59) | -1.07 (-1.44, -0.7) | 7946 (4789,12139) | 4.58 (2.73,7.09) | -1.08 (-1.44, -0.71) |
| Rwanda | 13515 (10974,16711) | 104.44 (85.07,128.12) | -4.03 (-5.86, -2.17) | 6414 (4021,11406) | 68.03 (42.81,123.22) | -1.12 (-2.48, 0.26) | 211 (122,365) | 2.2 (1.26,3.78) | -1.25 (-2.61, 0.13) |
| Saint Kitts and Nevis | 118 (91,152) | 209.69 (162.41,268.28) | 0.26 (0.11, 0.41) | 36 (31,43) | 59.85 (50.01,72.19) | 0.14 (0.03, 0.26) | 1 (1,2) | 2.02 (1.16,3.22) | 0.14 (0.02, 0.26) |
| Saint Lucia | 297 (236,380) | 175.36 (138.12,222.15) | 0.06 (-0.07, 0.19) | 99 (86,115) | 50.03 (42.34,59.72) | -0.06 (-0.15, 0.03) | 3 (2,5) | 1.69 (1,2.67) | -0.06 (-0.16, 0.04) |
| Saint Vincent and the Grenadines | 207 (163,263) | 184.44 (145.97,234.91) | 0.2 (0.05, 0.36) | 65 (56,77) | 53.31 (45.34,63.13) | 0.22 (0.1, 0.34) | 2 (1,3) | 1.8 (1.06,2.76) | 0.21 (0.09, 0.33) |
| Samoa | 282 (224,354) | 137.02 (109.53,173.29) | 0.07 (-0.68, 0.82) | 83 (70,98) | 47.95 (41.12,55.27) | 0.24 (-0.18, 0.66) | 3 (2,4) | 1.6 (0.96,2.46) | 0.22 (-0.21, 0.65) |
| San Marino | 99 (72,134) | 311.9 (224.19,430.72) | -0.06 (-0.18, 0.05) | 56 (48,64) | 109.81 (92.49,128.7) | -0.01 (-0.12, 0.1) | 2 (1,3) | 3.68 (2.17,5.64) | -0.02 (-0.13, 0.09) |
| Sao Tome and Principe | 353 (276,446) | 167.57 (131.13,213.8) | 0.42 (0.34, 0.5) | 87 (72,105) | 53.1 (44.73,61.89) | 0.57 (0.52, 0.61) | 3 (2,5) | 1.78 (1.06,2.73) | 0.55 (0.5, 0.59) |
| Saudi Arabia | 239890 (186788,308565) | 550.53 (426.39,707.82) | 0.47 (0.38, 0.55) | 61011 (50016,73887) | 170.71 (144.87,201.57) | 0.48 (0.42, 0.54) | 2083 (1216,3311) | 5.71 (3.42,8.94) | 0.47 (0.42, 0.53) |
| Senegal | 16133 (12833,20454) | 104.6 (83.61,131.52) | -0.35 (-0.5, -0.19) | 4085 (3408,4884) | 35.79 (30.88,41.3) | -0.07 (-0.14, 0.01) | 139 (82,216) | 1.19 (0.73,1.8) | -0.09 (-0.17, -0.01) |
| Serbia | 33410 (25510,43317) | 398.91 (302.76,525.97) | -0.91 (-1.36, -0.45) | 12509 (10799,14407) | 112.72 (93.36,134.98) | -0.68 (-0.95, -0.41) | 416 (250,644) | 3.8 (2.27,5.96) | -0.7 (-0.98, -0.42) |
| Seychelles | 143 (116,180) | 134.99 (109.11,168.37) | -0.45 (-0.56, -0.35) | 47 (40,55) | 41.64 (35.74,49.06) | -0.43 (-0.49, -0.37) | 2 (1,2) | 1.4 (0.84,2.19) | -0.44 (-0.5, -0.38) |
| Sierra Leone | 9455 (7670,11621) | 106.4 (85.91,130.2) | -3.16 (-4.51, -1.79) | 2651 (2135,3325) | 41.31 (34.32,51.02) | -1.26 (-2.04, -0.48) | 90 (56,136) | 1.37 (0.87,2.04) | -1.35 (-2.15, -0.53) |
| Singapore | 12439 (9167,16679) | 240.47 (176.57,328.08) | -0.67 (-0.81, -0.53) | 5640 (4919,6512) | 82.81 (69.89,98.68) | -0.61 (-0.77, -0.45) | 189 (112,291) | 2.79 (1.63,4.35) | -0.61 (-0.77, -0.45) |
| Slovakia | 28159 (21101,36843) | 525.14 (396.97,693.19) | -0.62 (-0.66, -0.58) | 10639 (9158,12437) | 153.34 (128.49,184.6) | -0.69 (-0.76, -0.62) | 352 (211,546) | 5.15 (3.04,8.16) | -0.68 (-0.75, -0.62) |
| Slovenia | 12567 (9491,16561) | 579.96 (439.95,768.98) | -0.5 (-0.76, -0.23) | 5687 (4853,6587) | 175.92 (146.18,210.11) | -0.55 (-0.78, -0.32) | 185 (110,276) | 5.87 (3.5,9.23) | -0.55 (-0.78, -0.32) |
| Solomon Islands | 1472 (1134,1919) | 235.19 (180.08,306.39) | 0.56 (0.45, 0.66) | 416 (348,492) | 89.4 (77.08,103.77) | 0.55 (0.49, 0.61) | 14 (8,22) | 2.96 (1.77,4.53) | 0.53 (0.47, 0.6) |
| Somalia | 35933 (27499,47842) | 158.65 (123.37,203.09) | 0.26 (-0.77, 1.3) | 8140 (6081,10912) | 51.54 (41.44,65.87) | 0.14 (-0.39, 0.67) | 278 (168,444) | 1.72 (1.09,2.67) | 0.14 (-0.41, 0.69) |
| South Africa | 84703 (68359,104367) | 142.98 (115.42,176.1) | -1.54 (-1.67, -1.42) | 26594 (23040,31013) | 48.17 (42.18,55.48) | -1.66 (-1.81, -1.51) | 895 (550,1360) | 1.61 (0.99,2.43) | -1.67 (-1.82, -1.52) |
| South Sudan | 14336 (11250,18208) | 138.15 (109.44,172.08) | 0.8 (-0.5, 2.12) | 3547 (2665,4673) | 46.51 (36.83,60.22) | 0.64 (-0.08, 1.36) | 120 (74,191) | 1.54 (0.97,2.38) | 0.65 (-0.1, 1.4) |
| Spain | 135225 (95161,185861) | 311.04 (218.3,433.57) | -0.43 (-0.55, -0.3) | 75746 (66078,86689) | 111.82 (94.6,134.12) | -0.24 (-0.37, -0.11) | 2484 (1491,3775) | 3.75 (2.21,5.81) | -0.26 (-0.39, -0.13) |
| Sri Lanka | 45796 (35540,59451) | 201.75 (156.57,260.57) | -2.24 (-3.33, -1.14) | 19060 (15818,23858) | 79.67 (65.8,99.47) | -0.62 (-1.2, -0.04) | 628 (410,933) | 2.62 (1.7,3.91) | -0.72 (-1.32, -0.12) |
| Sudan | 91738 (74687,112792) | 199.96 (163.43,246.05) | -1.3 (-1.72, -0.87) | 22959 (18708,27660) | 64.21 (54.4,75.82) | -0.73 (-0.97, -0.49) | 781 (483,1203) | 2.15 (1.34,3.26) | -0.76 (-1.01, -0.51) |
| Suriname | 937 (757,1178) | 163.95 (132.06,206.46) | 0.2 (0.12, 0.29) | 304 (261,358) | 50.92 (43.46,59.78) | 0.12 (0.05, 0.19) | 10 (6,16) | 1.71 (1.02,2.72) | 0.11 (0.04, 0.18) |
| Sweden | 34296 (24056,47082) | 294.21 (208.71,406.91) | -0.68 (-0.83, -0.52) | 19011 (15951,22153) | 112.29 (94.19,134.02) | -0.62 (-0.78, -0.47) | 615 (368,933) | 3.72 (2.19,5.79) | -0.64 (-0.8, -0.48) |
| Switzerland | 39897 (27782,55174) | 412.96 (284.94,575.12) | -1.36 (-1.53, -1.18) | 22369 (19136,26100) | 156.06 (131.77,186.16) | -1.3 (-1.42, -1.19) | 723 (439,1080) | 5.18 (3.06,7.82) | -1.3 (-1.43, -1.18) |
| Syrian Arab Republic | 34646 (26153,45242) | 242.88 (185.59,317.85) | 5.11 (3.27, 6.98) | 13433 (9344,20441) | 101.14 (69.22,158.73) | 4.08 (2.89, 5.29) | 445 (281,730) | 3.33 (2.1,5.47) | 4.11 (2.89, 5.34) |
| Taiwan (Province of China) | 29428 (22654,38294) | 112.34 (88.58,142.92) | -2.63 (-2.87, -2.38) | 13415 (11795,15196) | 39.57 (34.45,45.58) | -2.43 (-2.6, -2.25) | 446 (263,671) | 1.33 (0.78,2.03) | -2.44 (-2.62, -2.26) |
| Tajikistan | 22652 (18018,28523) | 211.42 (168.33,266.3) | -2.63 (-3.57, -1.68) | 5780 (4660,6995) | 63.42 (52.61,75.58) | -1.99 (-2.59, -1.39) | 197 (122,304) | 2.15 (1.33,3.25) | -2.02 (-2.63, -1.41) |
| Thailand | 126946 (100826,159663) | 188.94 (151.59,237.1) | -0.59 (-0.74, -0.45) | 50782 (44982,57663) | 59.85 (51.21,69.52) | -0.72 (-0.82, -0.62) | 1691 (1022,2526) | 2.01 (1.2,3.06) | -0.72 (-0.82, -0.62) |
| Timor-Leste | 1808 (1448,2216) | 129.05 (104.21,158.07) | -4.98 (-6.44, -3.5) | 701 (502,1067) | 66.4 (46.41,104.21) | -1.96 (-2.66, -1.26) | 23 (15,37) | 2.17 (1.36,3.47) | -2.1 (-2.82, -1.37) |
| Togo | 9291 (7593,11445) | 115.12 (94.52,141.77) | -0.3 (-0.49, -0.11) | 2372 (1991,2808) | 39.93 (34.65,46) | -0.17 (-0.26, -0.08) | 81 (49,127) | 1.33 (0.8,2.08) | -0.17 (-0.27, -0.08) |
| Tokelau | 2 (1,2) | 123.28 (96.63,158.29) | -0.12 (-0.19, -0.06) | 1 (1,1) | 41.77 (35.88,48.78) | -0.12 (-0.18, -0.06) | 0 (0,0) | 1.39 (0.85,2.14) | -0.13 (-0.19, -0.07) |
| Tonga | 108 (85,138) | 103.53 (81.26,131.42) | -0.42 (-0.64, -0.19) | 32 (27,38) | 34.27 (29.54,40.07) | -0.39 (-0.51, -0.26) | 1 (1,2) | 1.14 (0.68,1.77) | -0.4 (-0.53, -0.27) |
| Trinidad and Tobago | 2362 (1941,2913) | 183.36 (149.32,227.4) | 0.44 (0.25, 0.63) | 786 (689,908) | 51.82 (44.17,61.01) | 0.22 (0.09, 0.35) | 26 (16,40) | 1.75 (1.05,2.72) | 0.22 (0.09, 0.35) |
| Tunisia | 26317 (21072,32959) | 225.74 (179.96,283.05) | -0.48 (-0.54, -0.41) | 8141 (6924,9512) | 65.76 (55.18,77.74) | -0.47 (-0.52, -0.41) | 273 (162,423) | 2.21 (1.31,3.44) | -0.49 (-0.54, -0.43) |
| Turkmenistan | 11653 (9072,14777) | 219.16 (170.91,278.16) | -0.86 (-1.06, -0.65) | 2915 (2396,3578) | 58.29 (48.47,70.77) | -0.89 (-1.02, -0.76) | 100 (59,161) | 1.98 (1.17,3.17) | -0.89 (-1.02, -0.76) |
| Tuvalu | 17 (14,22) | 140.53 (113.38,175.33) | -0.47 (-0.63, -0.31) | 5 (5,6) | 48.5 (42.13,56.14) | -0.46 (-0.6, -0.31) | 0 (0,0) | 1.62 (0.96,2.5) | -0.46 (-0.61, -0.32) |
| Türkiye | 156205 (122675,201356) | 189.6 (148.53,243.88) | -0.51 (-0.67, -0.35) | 50127 (42915,58364) | 58.16 (49.48,68.3) | -0.09 (-0.2, 0.01) | 1676 (1013,2637) | 1.94 (1.16,3.07) | -0.14 (-0.25, -0.04) |
| Uganda | 46165 (37281,57056) | 107.01 (87.18,131.72) | -0.68 (-1.04, -0.32) | 11757 (9492,14563) | 42.46 (35.35,53.18) | -0.59 (-0.76, -0.42) | 399 (242,618) | 1.4 (0.9,2.14) | -0.59 (-0.76, -0.41) |
| Ukraine | 196208 (154775,253412) | 486.82 (387.76,623.52) | -1.06 (-1.23, -0.88) | 73466 (63792,85051) | 138.71 (116.7,164.26) | -1.21 (-1.4, -1.02) | 2449 (1475,3771) | 4.68 (2.76,7.25) | -1.19 (-1.38, -1.01) |
| United Arab Emirates | 30541 (24079,38332) | 286.32 (229.36,357.41) | -0.28 (-0.31, -0.26) | 8772 (7353,10365) | 83.81 (70.59,99.1) | -0.42 (-0.45, -0.39) | 300 (177,475) | 2.83 (1.67,4.4) | -0.41 (-0.44, -0.39) |
| United Kingdom | 198023 (138804,273219) | 277.37 (194.98,383.65) | -0.34 (-0.51, -0.18) | 105750 (91132,122477) | 106.04 (89.89,125.89) | -0.17 (-0.33, -0.01) | 3448 (2112,5166) | 3.52 (2.1,5.38) | -0.19 (-0.35, -0.03) |
| United Republic of Tanzania | 62965 (50569,78442) | 107.57 (87.35,134.59) | -0.23 (-0.26, -0.19) | 15286 (12690,18371) | 36.15 (31.35,41.8) | -0.11 (-0.14, -0.08) | 521 (306,816) | 1.2 (0.72,1.87) | -0.11 (-0.14, -0.08) |
| United States of America | 1036142 (767870,1365875) | 264.92 (200.18,341.52) | -0.76 (-0.95, -0.57) | 571658 (490281,662225) | 114.18 (98.07,132.33) | -0.2 (-0.34, -0.05) | 18422 (11360,27526) | 3.73 (2.29,5.61) | -0.26 (-0.41, -0.12) |
| United States Virgin Islands | 146 (117,184) | 173.46 (138.73,222.5) | -0.52 (-0.72, -0.33) | 62 (55,70) | 52.47 (44.84,62.12) | -0.48 (-0.64, -0.32) | 2 (1,3) | 1.76 (1.06,2.79) | -0.51 (-0.67, -0.34) |
| Uruguay | 10732 (8142,14338) | 315.96 (237.45,423.16) | -0.41 (-0.46, -0.36) | 4482 (3897,5154) | 104.65 (88.37,124.85) | -0.28 (-0.32, -0.25) | 148 (88,227) | 3.52 (2.1,5.43) | -0.29 (-0.33, -0.25) |
| Uzbekistan | 83045 (67029,103678) | 238.58 (192.92,298.65) | -0.78 (-0.91, -0.65) | 21355 (17854,25784) | 65.36 (55.2,78.27) | -0.68 (-0.81, -0.55) | 728 (431,1139) | 2.22 (1.31,3.46) | -0.69 (-0.82, -0.56) |
| Vanuatu | 381 (309,470) | 123.5 (99.73,151.99) | -0.17 (-0.45, 0.12) | 104 (88,122) | 42.08 (36.48,48.4) | -0.08 (-0.23, 0.07) | 4 (2,5) | 1.41 (0.84,2.15) | -0.1 (-0.25, 0.06) |
| Venezuela (Bolivarian Republic of) | 67479 (53951,86935) | 265.85 (211.52,341.4) | -0.64 (-0.97, -0.31) | 21596 (18621,25380) | 78.94 (67.46,94.09) | -0.56 (-0.75, -0.37) | 726 (442,1111) | 2.66 (1.6,4.13) | -0.56 (-0.75, -0.37) |
| Viet Nam | 191422 (152945,239922) | 193.43 (153.99,241.74) | 0.47 (0.38, 0.56) | 65258 (56111,76154) | 68.76 (59.19,79.92) | 0.66 (0.56, 0.77) | 2187 (1302,3396) | 2.29 (1.36,3.56) | 0.65 (0.55, 0.75) |
| Yemen | 163576 (106511,254280) | 432.44 (295.17,657.96) | 2.17 (1.4, 2.94) | 33276 (23221,47852) | 106.87 (80.83,146.13) | 1.08 (0.64, 1.53) | 1142 (634,1986) | 3.62 (2.09,6.03) | 1.12 (0.66, 1.58) |
| Zambia | 20970 (16985,26184) | 114.61 (93.02,143.46) | -0.09 (-0.16, -0.01) | 4997 (4128,6038) | 39.66 (34.76,45.96) | 0.03 (-0.03, 0.09) | 171 (99,270) | 1.32 (0.79,2.01) | 0.02 (-0.04, 0.08) |
| Zimbabwe | 17174 (14131,20885) | 113.25 (93.02,137.04) | -0.31 (-0.42, -0.19) | 4316 (3607,5135) | 38.63 (33.61,44.34) | -0.31 (-0.39, -0.22) | 147 (86,229) | 1.29 (0.78,1.97) | -0.31 (-0.4, -0.22) |

EAPC Estimated Annual Percentage Change, YLDs Years Lived with Disability, ASR Age-Standardized Rate, UI uncertainty interval, CI confidence interval.
