# Supplementary material for: Global, regional, and national burden of clavicle, scapula, or humerus fracture in 204 countries and territories, 1990 to 2021: A systematic analysis from the Global Burden of Disease Study 2021
Source: Medicine (Baltimore). 2026 May 22;105(21):e48862. doi: 10.1097/MD.0000000000048862 (PMC13201055; doi:10.1097/MD.0000000000048862)
Supplement: Supplementary file 4 [file medi-105-e48862-s004.docx]

**Supplementary table 2.** Global Incidence, prevalence and YLDs of Fracture of clavicle, scapula, or humerus and their EAPC from 1990 to 2021 by age groups.

| **Age Groups** | **Incidence** |  |  |  |  | **Prevalence** |  |  |  |  | **YLDs** |  |  |  |  |
| --- | --- | --- | --- | --- | --- | --- | --- | --- | --- | --- | --- | --- | --- | --- | --- |
|  | **Number** |  | **ASR per 100,000** |  |  | **Number** |  | **ASR per 100,000** |  |  | **Number** |  | **ASR per 100,000** |  |  |
|  | **1990** | **2021** | **1990** | **2021** | **Estimated Annual Percentage Change (95% CI)** | **1990** | **2021** | **1990** | **2021** | **Estimated Annual Percentage Change (95% CI)** | **1990** | **2021** | **1990** | **2021** | **Estimated Annual Percentage Change (95% CI)** |
| **<5** | **1411353 (1124353,1772360)** | **841864 (665651,1065339)** | **227.66 (181.37,285.89)** | **127.91 (101.14,161.86)** | **-1.91 (-2.03, -1.79)** | **208007 (150355,290435)** | **123767 (88648,172469)** | **33.55 (24.25,46.85)** | **18.8 (13.47,26.2)** | **-1.92 (-2.04, -1.8)** | **7262 (3932,12250)** | **4321 (2341,7374)** | **1.17 (0.63,1.98)** | **0.66 (0.36,1.12)** | **-1.92 (-2.04, -1.8)** |
| **5-9** | **1429495 (1025249,1935449)** | **1153937 (824947,1583582)** | **244.97 (175.7,331.68)** | **167.95 (120.07,230.49)** | **-1.1 (-1.19, -1.01)** | **223395 (155787,316089)** | **177286 (120329,253285)** | **38.28 (26.7,54.17)** | **25.8 (17.51,36.87)** | **-1.17 (-1.25, -1.08)** | **7796 (4219,13819)** | **6185 (3271,10987)** | **1.34 (0.72,2.37)** | **0.9 (0.48,1.6)** | **-1.17 (-1.25, -1.09)** |
| **10-14** | **1345525 (963302,1811552)** | **1239873 (868830,1705775)** | **251.18 (179.83,338.18)** | **185.99 (130.33,255.88)** | **-1.05 (-1.11, -0.98)** | **225669 (159820,314147)** | **202756 (143778,286651)** | **42.13 (29.83,58.64)** | **30.41 (21.57,43)** | **-1.14 (-1.2, -1.07)** | **7867 (4268,13278)** | **7067 (3801,12183)** | **1.47 (0.8,2.48)** | **1.06 (0.57,1.83)** | **-1.14 (-1.2, -1.07)** |
| **15-19** | **1761355 (1329096,2296164)** | **1571181 (1168632,2064275)** | **339.1 (255.88,442.06)** | **251.8 (187.29,330.82)** | **-0.96 (-1.06, -0.85)** | **305833 (231198,401566)** | **266713 (198106,359142)** | **58.88 (44.51,77.31)** | **42.74 (31.75,57.56)** | **-1.05 (-1.15, -0.96)** | **10654 (6005,17606)** | **9300 (5161,15468)** | **2.05 (1.16,3.39)** | **1.49 (0.83,2.48)** | **-1.05 (-1.14, -0.95)** |
| **20-24** | **1720313 (1303489,2221462)** | **1601300 (1210577,2076202)** | **349.59 (264.89,451.44)** | **268.15 (202.72,347.68)** | **-0.96 (-1.03, -0.88)** | **330236 (250637,416924)** | **297161 (225781,382438)** | **67.11 (50.93,84.73)** | **49.76 (37.81,64.04)** | **-1.08 (-1.15, -1.01)** | **11455 (6698,18246)** | **10328 (6013,16718)** | **2.33 (1.36,3.71)** | **1.73 (1.01,2.8)** | **-1.07 (-1.14, -1)** |
| **25-29** | **1388350 (1063085,1836294)** | **1438728 (1085001,1935829)** | **313.67 (240.18,414.87)** | **244.54 (184.42,329.03)** | **-0.83 (-0.89, -0.77)** | **308221 (240090,392237)** | **304652 (235281,397040)** | **69.64 (54.24,88.62)** | **51.78 (39.99,67.48)** | **-1.03 (-1.08, -0.97)** | **10639 (6078,16797)** | **10535 (5980,16512)** | **2.4 (1.37,3.79)** | **1.79 (1.02,2.81)** | **-1.01 (-1.07, -0.96)** |
| **30-34** | **1147705 (867420,1546970)** | **1420520 (1071983,1974516)** | **297.78 (225.06,401.37)** | **235 (177.34,326.65)** | **-0.83 (-0.87, -0.79)** | **289237 (235509,360552)** | **342601 (274594,433045)** | **75.04 (61.1,93.55)** | **56.68 (45.43,71.64)** | **-1.03 (-1.07, -0.99)** | **9959 (5848,15693)** | **11790 (6926,18733)** | **2.58 (1.52,4.07)** | **1.95 (1.15,3.1)** | **-1.03 (-1.07, -0.99)** |
| **35-39** | **975466 (707988,1330568)** | **1270569 (909787,1727556)** | **276.93 (200.99,377.74)** | **226.54 (162.21,308.02)** | **-0.84 (-0.93, -0.76)** | **284421 (231337,344797)** | **354584 (287677,436263)** | **80.75 (65.68,97.89)** | **63.22 (51.29,77.78)** | **-1.01 (-1.09, -0.93)** | **9755 (5739,15308)** | **12148 (7143,19059)** | **2.77 (1.63,4.35)** | **2.17 (1.27,3.4)** | **-1.01 (-1.09, -0.93)** |
| **40-44** | **718568 (524514,965233)** | **1026625 (760497,1372822)** | **250.83 (183.09,336.93)** | **205.22 (152.02,274.43)** | **-0.83 (-0.91, -0.75)** | **253417 (216339,298885)** | **345411 (292853,410734)** | **88.46 (75.52,104.33)** | **69.05 (58.54,82.11)** | **-1 (-1.08, -0.91)** | **8634 (5085,13375)** | **11771 (7028,18219)** | **3.01 (1.78,4.67)** | **2.35 (1.4,3.64)** | **-1 (-1.08, -0.91)** |
| **45-49** | **538415 (404839,734754)** | **935260 (693301,1288354)** | **231.88 (174.35,316.44)** | **197.52 (146.42,272.09)** | **-0.65 (-0.73, -0.56)** | **226979 (195738,263339)** | **370702 (316065,435860)** | **97.75 (84.3,113.41)** | **78.29 (66.75,92.05)** | **-0.88 (-0.97, -0.79)** | **7690 (4582,11869)** | **12553 (7552,19198)** | **3.31 (1.97,5.11)** | **2.65 (1.6,4.05)** | **-0.88 (-0.97, -0.79)** |
| **50-54** | **498655 (364006,670969)** | **897815 (648174,1217194)** | **234.58 (171.24,315.64)** | **201.79 (145.68,273.57)** | **-0.47 (-0.53, -0.41)** | **246518 (216174,280926)** | **410166 (354808,471991)** | **115.97 (101.69,132.16)** | **92.19 (79.75,106.08)** | **-0.8 (-0.86, -0.74)** | **8303 (4891,12551)** | **13807 (8315,20927)** | **3.91 (2.3,5.9)** | **3.1 (1.87,4.7)** | **-0.8 (-0.86, -0.74)** |
| **55-59** | **420801 (305797,571101)** | **830303 (588434,1153798)** | **227.21 (165.12,308.37)** | **209.82 (148.7,291.56)** | **-0.21 (-0.24, -0.18)** | **251299 (223128,282652)** | **450213 (392161,514248)** | **135.69 (120.48,152.62)** | **113.77 (99.1,129.95)** | **-0.65 (-0.69, -0.62)** | **8396 (5091,12894)** | **15041 (9224,22832)** | **4.53 (2.75,6.96)** | **3.8 (2.33,5.77)** | **-0.65 (-0.69, -0.61)** |
| **60-64** | **367582 (261019,501055)** | **724707 (507844,1001149)** | **228.87 (162.52,311.97)** | **226.44 (158.68,312.81)** | **0 (-0.04, 0.04)** | **265568 (234377,303408)** | **458421 (400743,534151)** | **165.35 (145.93,188.91)** | **143.24 (125.21,166.9)** | **-0.55 (-0.61, -0.49)** | **8788 (5348,13393)** | **15174 (9225,22949)** | **5.47 (3.33,8.34)** | **4.74 (2.88,7.17)** | **-0.54 (-0.6, -0.49)** |
| **65-69** | **294769 (205895,407313)** | **666945 (452139,922146)** | **238.47 (166.57,329.52)** | **241.78 (163.91,334.3)** | **0.08 (0.05, 0.11)** | **252828 (220012,285980)** | **503427 (434533,578425)** | **204.54 (177.99,231.36)** | **182.51 (157.53,209.69)** | **-0.44 (-0.47, -0.41)** | **8255 (5018,12186)** | **16439 (9956,24716)** | **6.68 (4.06,9.86)** | **5.96 (3.61,8.96)** | **-0.43 (-0.46, -0.4)** |
| **70-74** | **233375 (157863,335948)** | **582863 (401766,845385)** | **275.66 (186.46,396.82)** | **283.16 (195.18,410.7)** | **-0.01 (-0.05, 0.04)** | **216088 (185558,247478)** | **496763 (419823,578644)** | **255.24 (219.18,292.32)** | **241.34 (203.96,281.11)** | **-0.33 (-0.39, -0.27)** | **6959 (4309,10342)** | **15986 (9811,23896)** | **8.22 (5.09,12.22)** | **7.77 (4.77,11.61)** | **-0.33 (-0.38, -0.27)** |
| **75-79** | **224528 (149628,329989)** | **493290 (325508,726419)** | **364.76 (243.08,536.08)** | **374.03 (246.81,550.8)** | **0.06 (-0.03, 0.15)** | **225880 (192835,266835)** | **446938 (377406,534845)** | **366.95 (313.27,433.49)** | **338.89 (286.16,405.54)** | **-0.27 (-0.34, -0.21)** | **7166 (4294,10734)** | **14163 (8610,21473)** | **11.64 (6.98,17.44)** | **10.74 (6.53,16.28)** | **-0.27 (-0.34, -0.21)** |
| **80-84** | **167819 (105897,247415)** | **443307 (278880,660046)** | **474.39 (299.35,699.39)** | **506.16 (318.42,753.62)** | **0.15 (0.09, 0.22)** | **190390 (150048,239067)** | **445209 (349253,562502)** | **538.19 (424.15,675.79)** | **508.33 (398.77,642.25)** | **-0.23 (-0.27, -0.19)** | **5936 (3586,8788)** | **13873 (8262,20672)** | **16.78 (10.14,24.84)** | **15.84 (9.43,23.6)** | **-0.23 (-0.27, -0.19)** |
| **85-89** | **85461 (54040,126293)** | **287911 (181995,434120)** | **565.55 (357.62,835.77)** | **629.7 (398.05,949.48)** | **0.23 (0.15, 0.32)** | **116769 (93091,148831)** | **348747 (275577,449064)** | **772.74 (616.04,984.91)** | **762.76 (602.73,982.17)** | **-0.11 (-0.17, -0.05)** | **3561 (2103,5331)** | **10642 (6259,16020)** | **23.57 (13.92,35.28)** | **23.27 (13.69,35.04)** | **-0.11 (-0.17, -0.05)** |
| **90-94** | **25060 (15696,38780)** | **123664 (78438,185129)** | **584.8 (366.28,904.98)** | **691.27 (438.46,1034.86)** | **0.5 (0.41, 0.59)** | **46646 (37668,58941)** | **199475 (160078,257491)** | **1088.54 (879.02,1375.45)** | **1115.04 (894.82,1439.35)** | **0.06 (-0.01, 0.13)** | **1389 (817,2099)** | **5946 (3495,9037)** | **32.41 (19.07,48.98)** | **33.24 (19.54,50.51)** | **0.07 (0, 0.14)** |
| **95+** | **5258 (3113,8857)** | **37863 (22833,61504)** | **516.46 (305.82,869.96)** | **694.69 (418.93,1128.44)** | **0.83 (0.68, 0.98)** | **14296 (11576,18403)** | **84914 (68067,109321)** | **1404.17 (1137.08,1807.64)** | **1557.96 (1248.87,2005.78)** | **0.24 (0.14, 0.34)** | **416 (245,638)** | **2467 (1459,3864)** | **40.82 (24.08,62.66)** | **45.27 (26.76,70.89)** | **0.24 (0.14, 0.34)** |

EAPC Estimated Annual Percentage Change, YLDs Years Lived with Disability, ASR Age-Standardized Rate, UI uncertainty interval, CI confidence interval.
