# Supplementary material for: Global, regional, and national burden of clavicle, scapula, or humerus fracture in 204 countries and territories, 1990 to 2021: A systematic analysis from the Global Burden of Disease Study 2021
Source: Medicine (Baltimore). 2026 May 22;105(21):e48862. doi: 10.1097/MD.0000000000048862 (PMC13201055; doi:10.1097/MD.0000000000048862)

**Supplementary figure 3.** Age-Standardized Rates of Incidence, Prevalence, and YLDs for Fracture of clavicle, scapula, or humerus in 2021, Including Their Estimated Annual Percentage Change (EAPC) from 1990 to 2021, for Different Age Groups. (A and B) Incidence. (C and D) Prevalence. (E and F) YLDs. YLDs Years Lived with Disability.


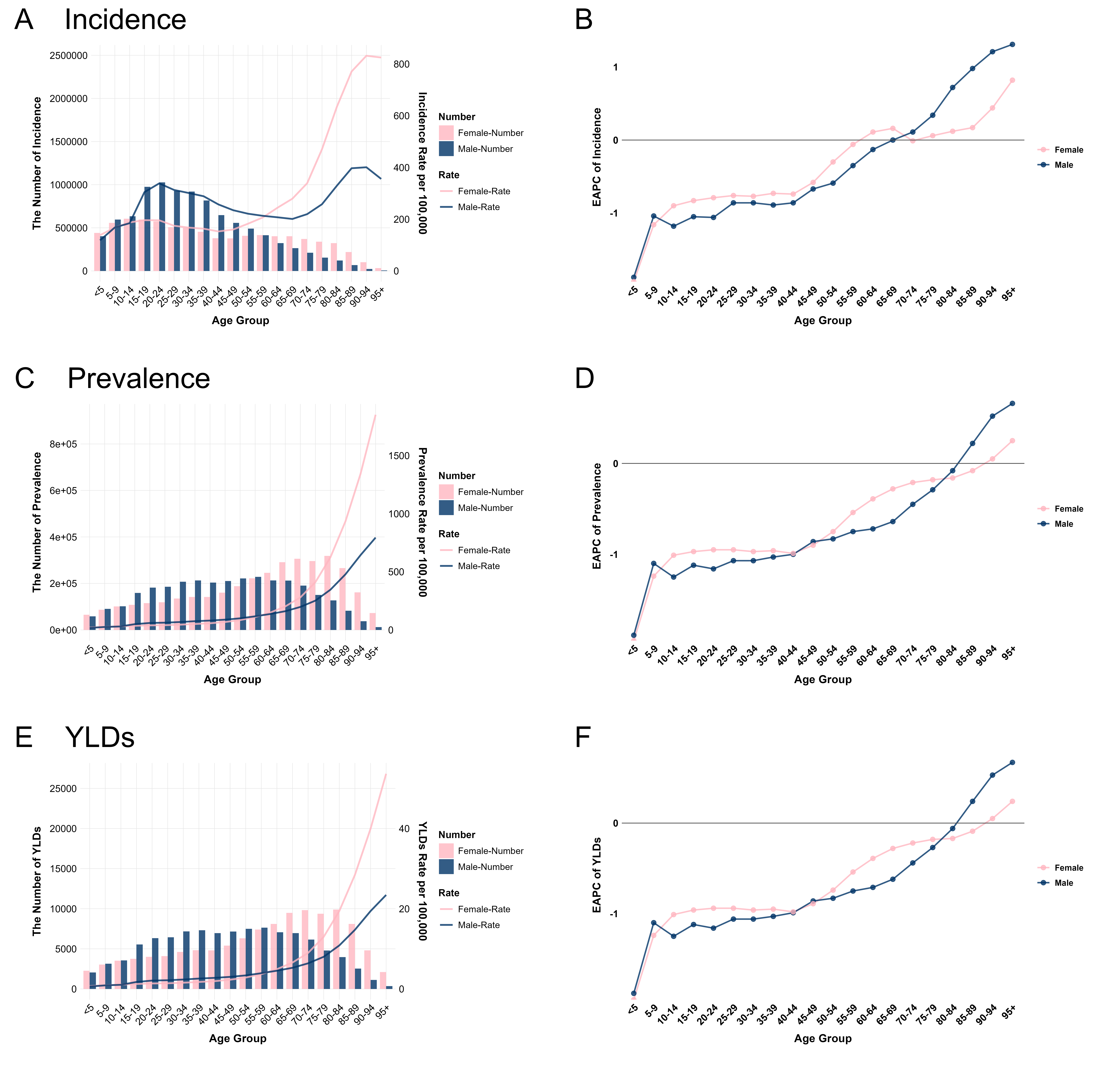

Supplement: Supplementary file 5 [file medi-105-e48862-s005.docx]
