# Supplementary material for: Global, regional, and national burden of clavicle, scapula, or humerus fracture in 204 countries and territories, 1990 to 2021: A systematic analysis from the Global Burden of Disease Study 2021
Source: Medicine (Baltimore). 2026 May 22;105(21):e48862. doi: 10.1097/MD.0000000000048862 (PMC13201055; doi:10.1097/MD.0000000000048862)
Supplement: Supplementary file 6 [file medi-105-e48862-s006.docx]

**Supplementary table 3.** The Slope Index of Inequality and the Concentration Index for Fracture of clavicle, scapula, or humerus from 1990 to 2021.

|  |  | **Incidence** | **Prevalence** | **YLDs** |
| --- | --- | --- | --- | --- |
| **Slope Index of Inequality (95% CI)** | **1990** | **237.79(181.820, 293.760)** | **80.860(62.398, 99.323)** | **2.729(2.111, 3.347)** |
|  | **2021** | **204.609(168.771, 240.446)** | **68.169(55.765, 80.573)** | **2.299(1.880, 2.717)** |
| **Concentration Index (95% CI)** | **1990** | **-0.064(-0.146, 0.022)** | **-0.166(-0.248, -0.092)** | **-0.161(-0.243, -0.086)** |
|  | **2021** | **-0.051(-0.136, 0.035)** | **-0.165(-0.255, -0.084)** | **-0.159(-0.249, -0.078)** |

YLDs Years Lived with Disability, CI confidence interval.
