# Supplementary material for: Global, regional, and national burden of clavicle, scapula, or humerus fracture in 204 countries and territories, 1990 to 2021: A systematic analysis from the Global Burden of Disease Study 2021
Source: Medicine (Baltimore). 2026 May 22;105(21):e48862. doi: 10.1097/MD.0000000000048862 (PMC13201055; doi:10.1097/MD.0000000000048862)
Supplement: Supplementary file 7 [file medi-105-e48862-s007.docx]

**Supplementary table 4.** Projection of the Global Burden of Fracture of clavicle, scapula, or humerus for the Next 15 Years Based on the BAPC Model.

| **Year** | **Incidence** | | **Prevalence** | | **YLDs** | |  |
| --- | --- | --- | --- | --- | --- | --- | --- |
|  | **Absolute numbers (95%UI)** | **ASR (per 100,000, 95%UI)** | **Absolute numbers (95%UI)** | **ASR (per 100,000, 95%UI)** | **Absolute numbers (95%UI)** | **ASR (per 100,000, 95%UI)** |  |
| **2022** | **17694374 (17311589,18077159)** | **219.93 (215.18,224.69)** | **6502018 (6397308,6606728)** | **80.82 (79.52,82.12)** | **215177 (211732,218622)** | **2.67 (2.63,2.72)** |  |
| **2023** | **17803154 (17080896,18525413)** | **219.18 (210.28,228.07)** | **6570835 (6385749,6755922)** | **80.89 (78.62,83.17)** | **217238 (211186,223289)** | **2.67 (2.6,2.75)** |  |
| **2024** | **17901252 (16753656,19048848)** | **218.34 (204.34,232.34)** | **6637337 (6350483,6924190)** | **80.96 (77.46,84.45)** | **219220 (209876,228564)** | **2.67 (2.56,2.79)** |  |
| **2025** | **17985726 (16344695,19626757)** | **217.4 (197.56,237.24)** | **6700452 (6294661,7106242)** | **80.99 (76.09,85.9)** | **221091 (207907,234275)** | **2.67 (2.51,2.83)** |  |
| **2026** | **18060637 (15866293,20254982)** | **216.41 (190.11,242.7)** | **6760981 (6220850,7301113)** | **81.01 (74.54,87.48)** | **222865 (205352,240378)** | **2.67 (2.46,2.88)** |  |
| **2027** | **18123878 (15321597,20926160)** | **215.34 (182.04,248.63)** | **6818278 (6129334,7507221)** | **81.01 (72.82,89.2)** | **224526 (202227,246825)** | **2.67 (2.4,2.93)** |  |
| **2028** | **18179530 (14719384,21639676)** | **214.23 (173.46,255.01)** | **6873853 (6022666,7725041)** | **81 (70.97,91.03)** | **226118 (198612,253624)** | **2.66 (2.34,2.99)** |  |
| **2029** | **18223034 (14060191,22385877)** | **213.05 (164.38,261.72)** | **6926586 (5900695,7952477)** | **80.98 (68.99,92.98)** | **227616 (194513,260720)** | **2.66 (2.27,3.05)** |  |
| **2030** | **18252628 (13346618,23158638)** | **211.77 (154.85,268.69)** | **6975462 (5763241,8187682)** | **80.93 (66.87,94.99)** | **228990 (189926,268053)** | **2.66 (2.2,3.11)** |  |
| **2031** | **18272545 (12585037,23960053)** | **210.44 (144.94,275.94)** | **7021503 (5611612,8431394)** | **80.86 (64.63,97.1)** | **230258 (184885,275631)** | **2.65 (2.13,3.17)** |  |
| **2032** | **18280701 (11776326,24785076)** | **209.04 (134.66,283.41)** | **7063955 (5445426,8682484)** | **80.77 (62.27,99.28)** | **231404 (179384,283425)** | **2.65 (2.05,3.24)** |  |
| **2033** | **18281120 (10926441,25635799)** | **207.61 (124.08,291.13)** | **7104528 (5266700,8942356)** | **80.68 (59.81,101.55)** | **232476 (173483,291468)** | **2.64 (1.97,3.31)** |  |
| **2034** | **18270437 (10036335,26504540)** | **206.11 (113.22,299)** | **7142230 (5075238,9209223)** | **80.57 (57.25,103.89)** | **233452 (167187,299717)** | **2.63 (1.89,3.38)** |  |
| **2035** | **18247551 (9108532,27386570)** | **204.54 (102.1,306.98)** | **7176057 (4870853,9481261)** | **80.44 (54.6,106.28)** | **234305 (160493,308117)** | **2.63 (1.8,3.45)** |  |
| **2036** | **18216332 (8147678,28284986)** | **202.94 (90.77,315.1)** | **7207046 (4654646,9759445)** | **80.29 (51.85,108.72)** | **235054 (153428,316680)** | **2.62 (1.71,3.53)** |  |

YLDs Years Lived with Disability, ASR Age-Standardized Rate, UI uncertainty interval.
